# Supplementary material for: The EF-hand domain of MINDY3 is a ubiquitin and RAD23 UBL-binding domain
Source: EMBO Rep. 2026 Jun 9;27(13):3604–31. doi: 10.1038/s44319-026-00825-1 (PMC13354579; doi:10.1038/s44319-026-00825-1)
Supplement: Supplementary file 1 — Appendix [file 44319_2026_825_MOESM1_ESM.pdf]

Appendix to “The EF-hand domain of MINDY3 is a Ubiquitin and RAD23 UBL-binding domain”

Table of Contents

APPENDIX FIGURE S1. MINDY3 PREFERENTIALLY BINDS LONG K48-LINKED UBIQUITIN CHAINS. 2

APPENDIX FIGURE S2. MINDY3EF-HAND IS A POLYUBIQUITIN BINDING MODULE. ... 3

APPENDIX FIGURE S3. LOSS OF MINDY3 DOES NOT IMPAIR NER, OR CLEARANCE OF UB^{G76V}-GFP IN CELLS. 5

Appendix Figure S1

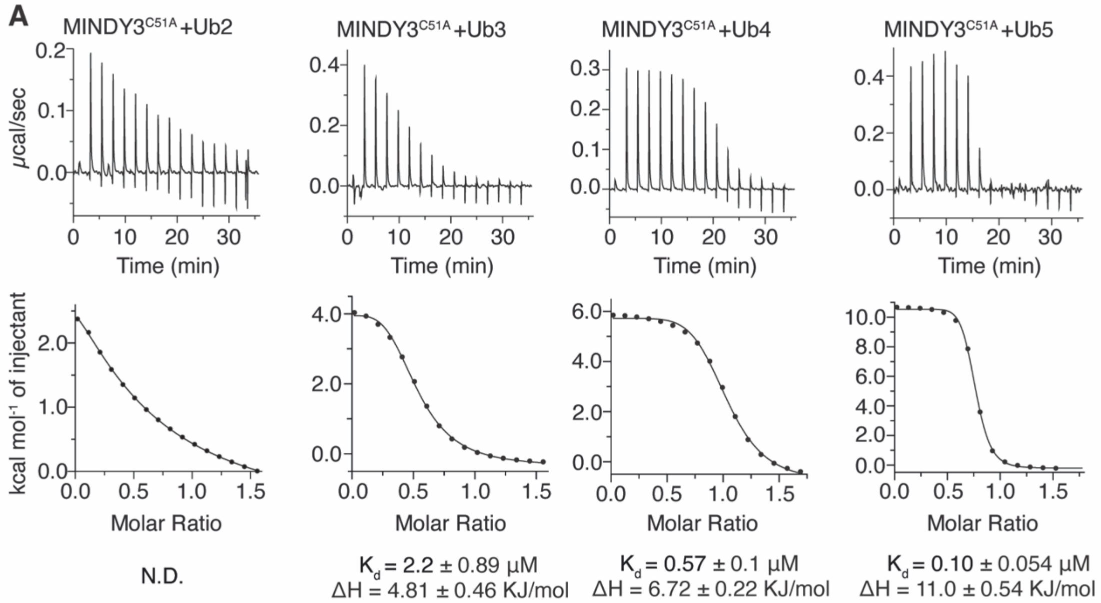

Appendix Figure S1. MINDY3 preferentially binds long K48-linked ubiquitin chains. (A) Isothermal Titration Calorimetry (ITC) measurements in which MINDY3^{C51A} was titrated into K48-linked polyUb chains of varying lengths.

Appendix Figure S2

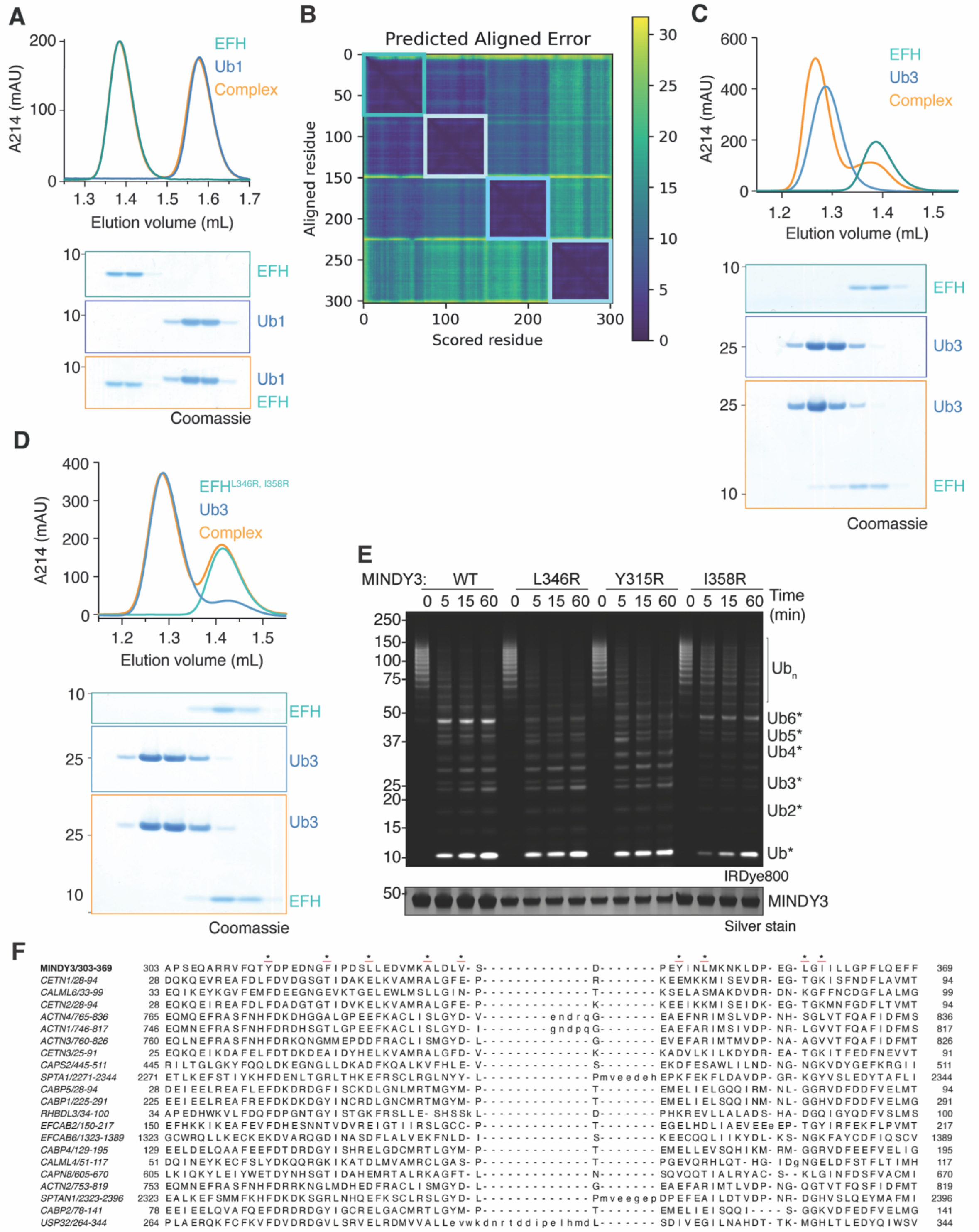

Appendix Figure S2. MINDY3$^{EF\text{-}hand}$ is a polyubiquitin binding module. (A) SEC of MINDY3$^{EF\text{-}hand}$ with monoub. **(B)** PAE plot for the AlphaFold model of MINDY3$^{EF\text{-}hand}$ + three ubiquitin molecules in figure 2D. Coloured boxes indicate individual proteins as in figure 2D. **(C-D)** SEC of K48-Ub3 with MINDY3$^{EF\text{-}hand}$ (C), or a mutant MINDY3$^{EF\text{-}hand}$ (D). **(E)** DUB assay monitoring cleavage of fluorescently labelled K48-linked

chains (Ub6-20) by MINDY3 WT and the EF hand mutants disrupting ubiquitin binding as site A (L346R), site B (Y315R) and site C (I358R). **(F)** Sequence alignment of the top 20 matches from a DALI search with MINDY3 EF hand against the human AlphaFold foldome. Also included is the EF-hand domain from USP32.

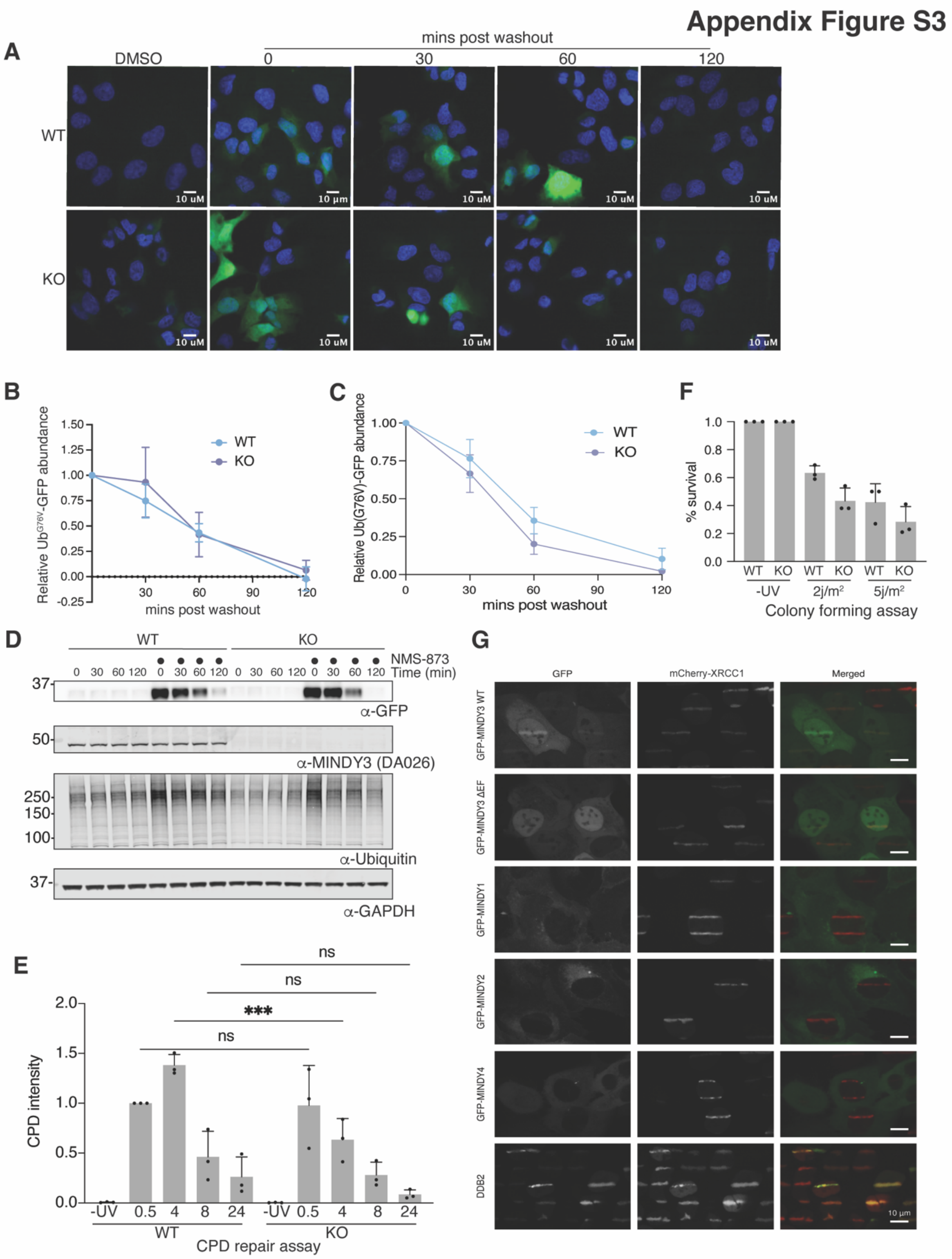

Appendix Figure S3. Loss of MINDY3 does not impair NER, or clearance of Ub^{G76V}-GFP in cells. (A) Representative images of Ub^{G76V}-GFP abundance in WT or MINDY3 depleted U2OS cells. To prevent the degradation of Ub^{G76V}-GFP cells were treated with the p97 inhibitor NMS-873 for 2 h. Degradation of Ub^{G76V}-GFP was assessed by fluorescence microscopy at the indicated times after NMS-873 washout.

(B) Quantification of the GFP signal detected in (A). Data points shown are mean nuclear GFP intensities normalised to 0% and 100% corresponding to DMSO and NMS treated samples respectively. Three independent experiments, with a minimum of 500 cells per condition were quantified. Error bars represent mean with standard deviation. **(C)** Relative stability of Ub^{G76V}-GFP in WT and MINDY3 KO U2OS cells. Cells were pre-treated with NMS-873 for 4 hours, and Ub^{G76V}-GFP abundance was then assessed at the indicated time by western blot and normalised for loading with corresponding GAPDH levels. Error bars indicate SD based on three independent experiments each with a minimum of two replicates. Error bars represent mean with standard deviation. **(D)** Representative western blot of data presented in (C). **(E)** U2OS, WT and MINDY3 KO cells were irradiated with UV-C (20 J/m^2), CPD abundance was monitored by dot blotting genomic DNA extracted at the indicated times and probing with a CPD and CDNA antibody. Quantification was performed on three biological replicates. Error bars represent mean with standard deviation. CPD signal was adjusted based on the cDNA signal and values were normalised to WT time 0.5 h. Statistical significance was assessed using an ordinary one-way Anova followed by Šídáks multiple comparisons. *** = >0.001, actual p value is 0.0004. **(F)** Quantification of colonies formed by U2OS cells, WT and MINDY3 KO after UV-C irradiation with the indicated doses. Quantification of four independent experiments performed in technical triplicates is shown. Error bars represent mean with standard deviation. **(G)** Representative images of U2OS cells co-expressing mCherry-XRCC1 together with either GFP-DDB2, GFP-MINDY3, GFP-MINDY3$^{\Delta EF\text{-}hand}$, GFP-MINDY1, GFP-MINDY2 or GFP-MINDY4 after microIR.
