# Supplementary material for: The EF-hand domain of MINDY3 is a ubiquitin and RAD23 UBL-binding domain
Source: EMBO Rep. 2026 Jun 9;27(13):3604–31. doi: 10.1038/s44319-026-00825-1 (PMC13354579; doi:10.1038/s44319-026-00825-1)
Supplement: Supplementary file 3 — Source data Fig. 1 [file 44319_2026_825_MOESM3_ESM.zip › Figure 1/1K/README.rtf]

Other mutants from source gel were excluded as they are not relevant to the point of the figure.
