# Supplementary figures and images for: The EF-hand domain of MINDY3 is a ubiquitin and RAD23 UBL-binding domain
Source: EMBO Rep. 2026 Jun 9;27(13):3604–31. doi: 10.1038/s44319-026-00825-1 (PMC13354579; doi:10.1038/s44319-026-00825-1)

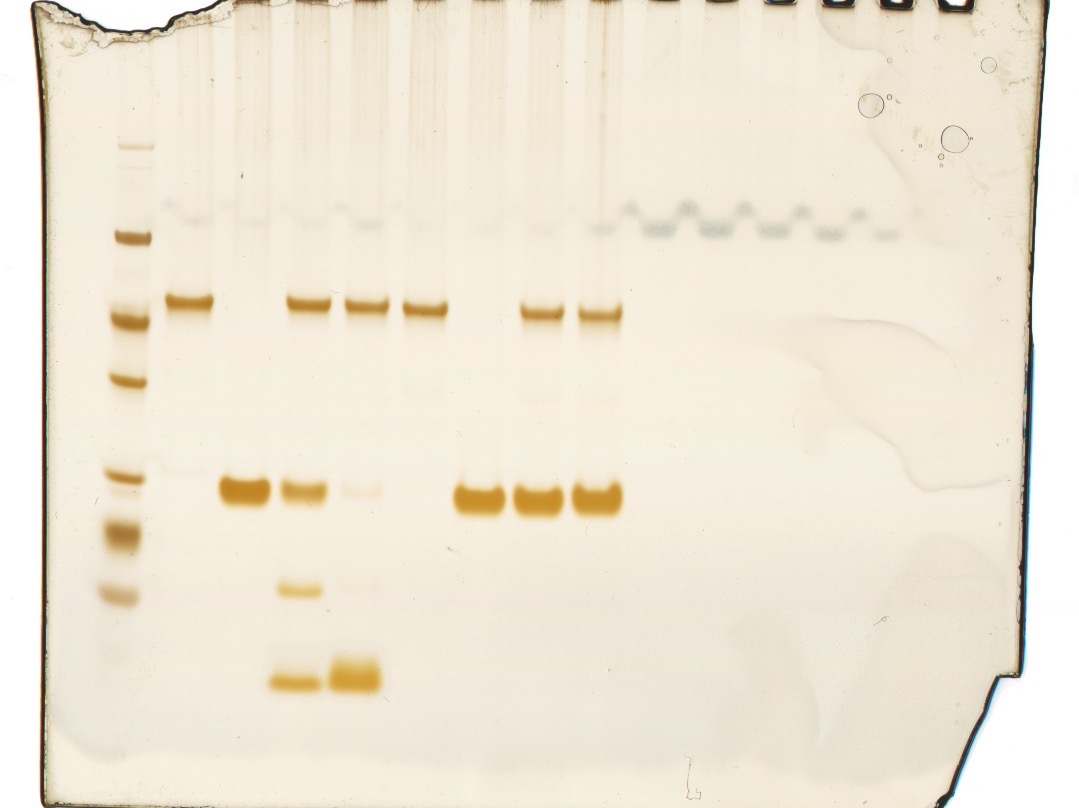

Supplement: Supplementary file 3 — Source data Fig. 1 [file 44319_2026_825_MOESM3_ESM.zip › Figure 1/1H/silver_stain_Q45A.jpeg]

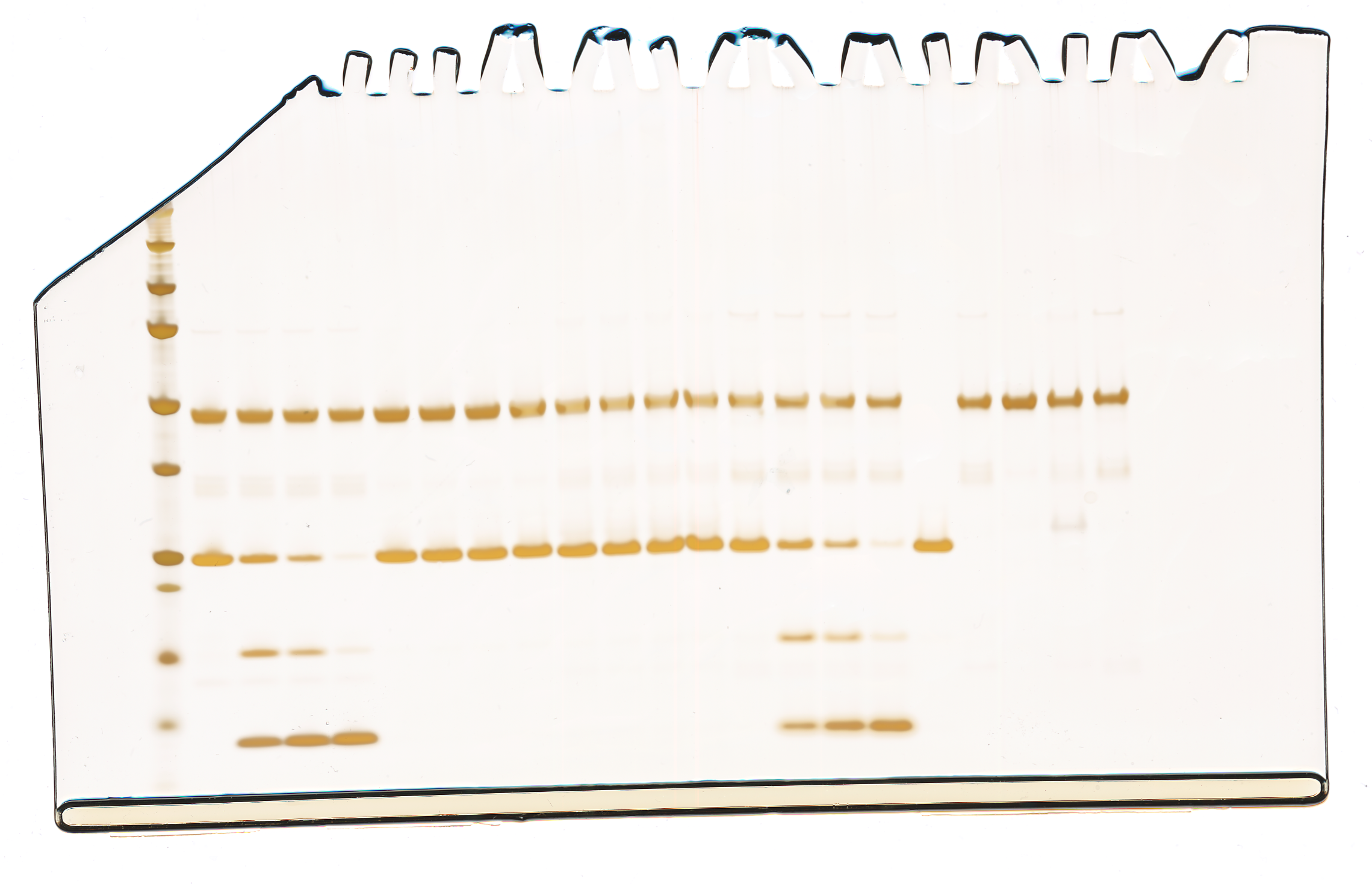

Supplement: Supplementary file 3 — Source data Fig. 1 [file 44319_2026_825_MOESM3_ESM.zip › Figure 1/1G/raw_gel.tif]

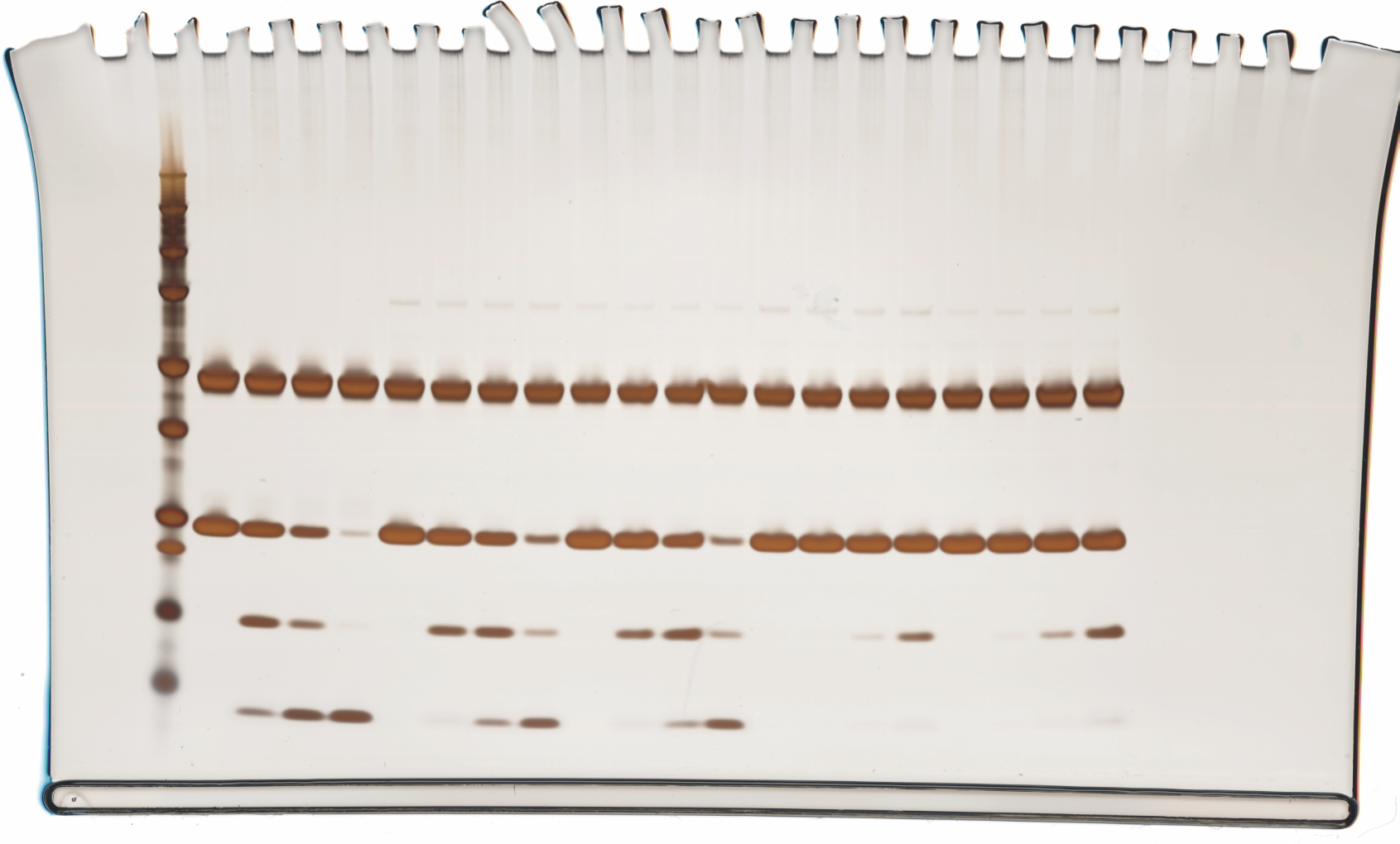

Supplement: Supplementary file 3 — Source data Fig. 1 [file 44319_2026_825_MOESM3_ESM.zip › Figure 1/1K/raw_gel.tif]

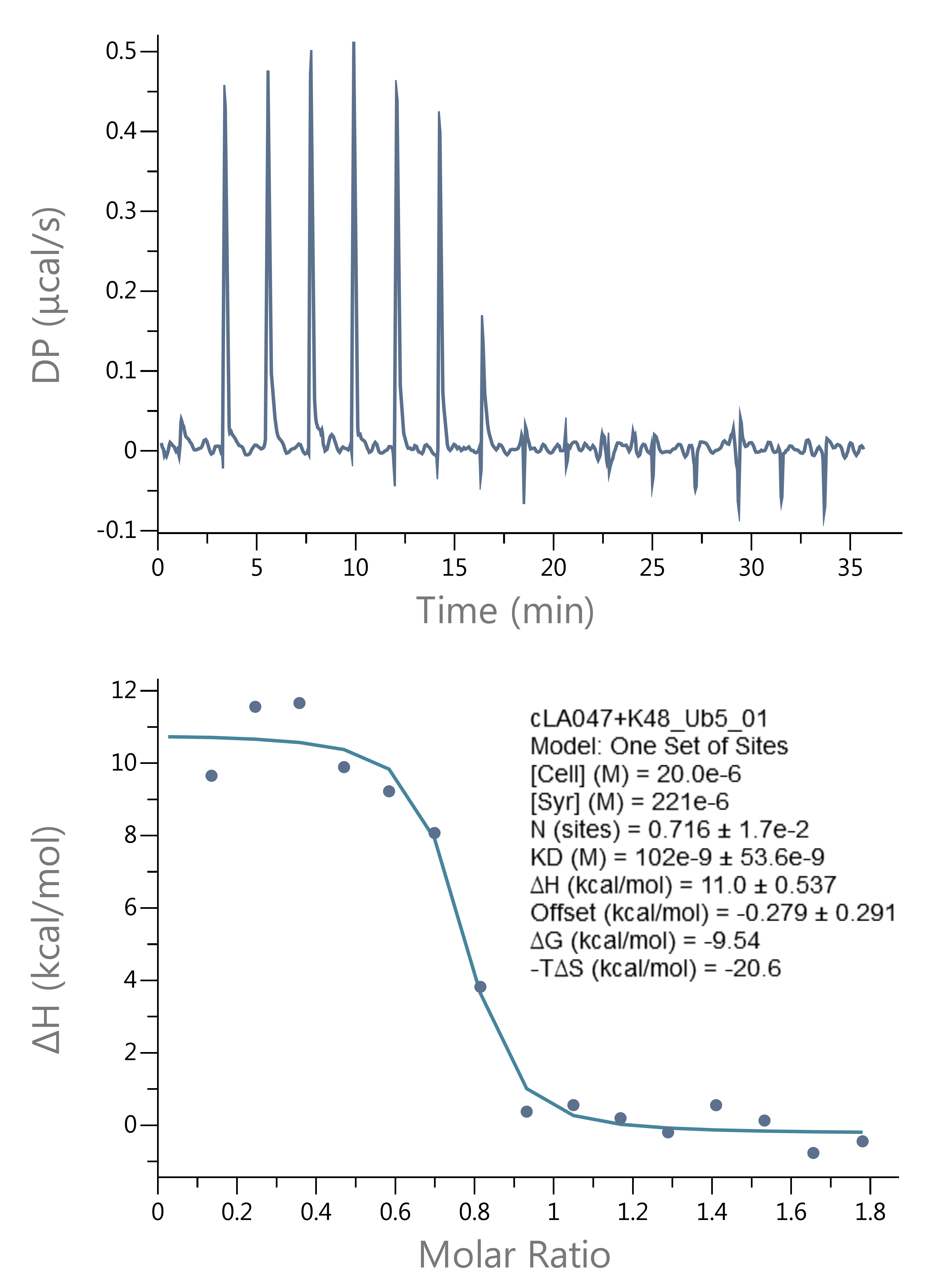

Supplement: Supplementary file 3 — Source data Fig. 1 [file 44319_2026_825_MOESM3_ESM.zip › Figure 1/1D/Ub5_raw_trace.bmp]

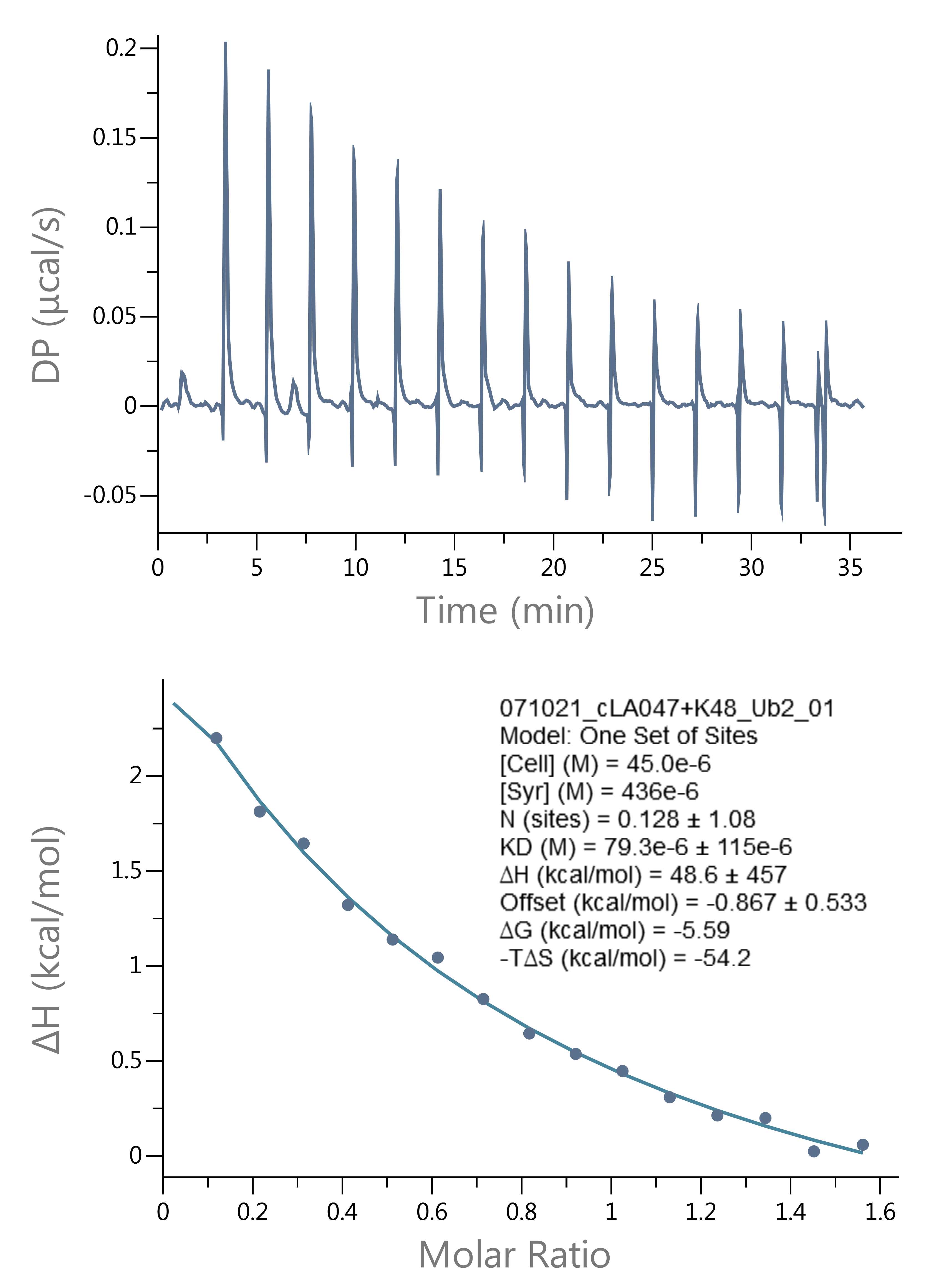

Supplement: Supplementary file 3 — Source data Fig. 1 [file 44319_2026_825_MOESM3_ESM.zip › Figure 1/1D/Ub2_raw_trace.bmp]

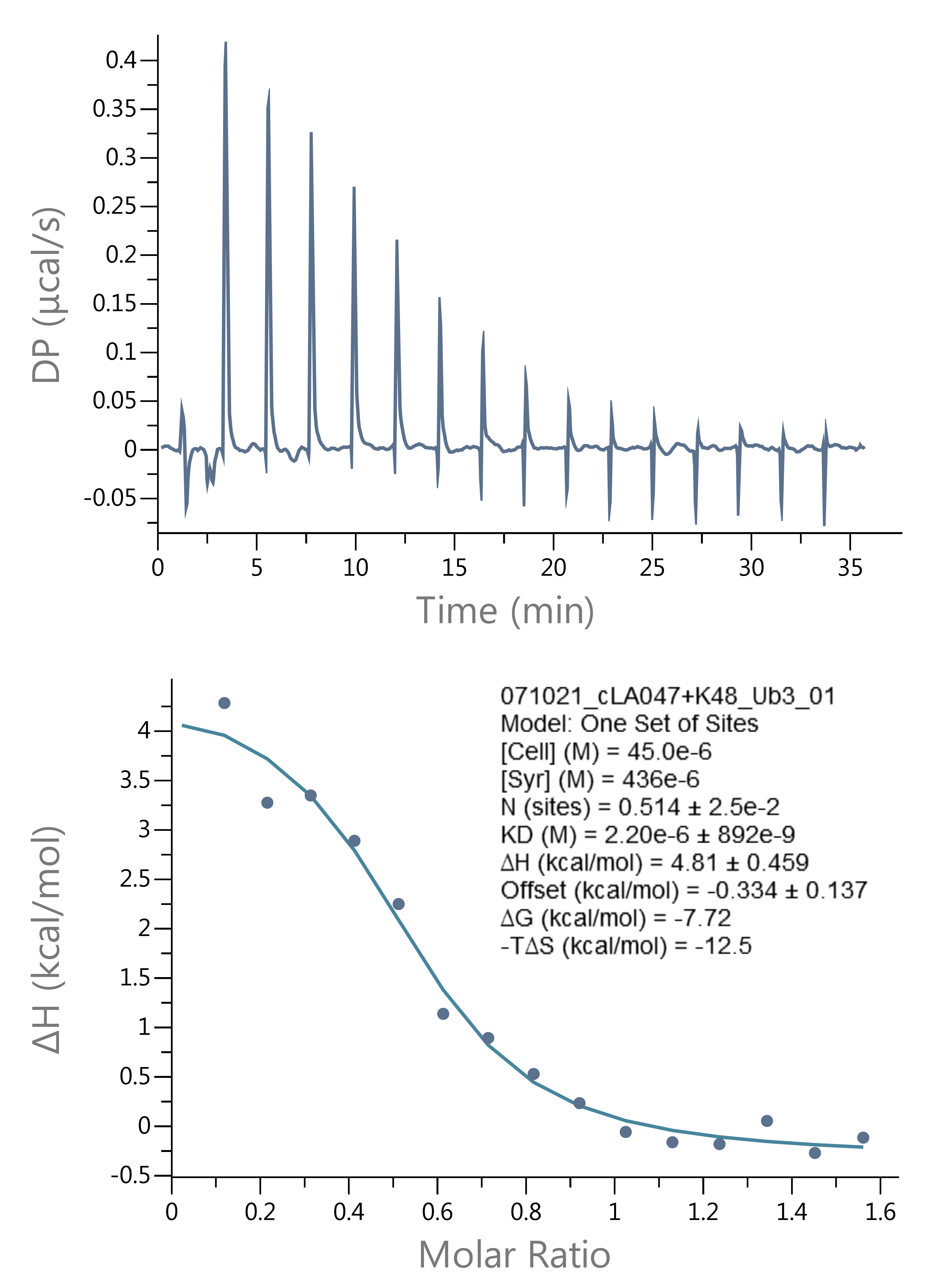

Supplement: Supplementary file 3 — Source data Fig. 1 [file 44319_2026_825_MOESM3_ESM.zip › Figure 1/1D/Ub3_raw_trace.bmp]

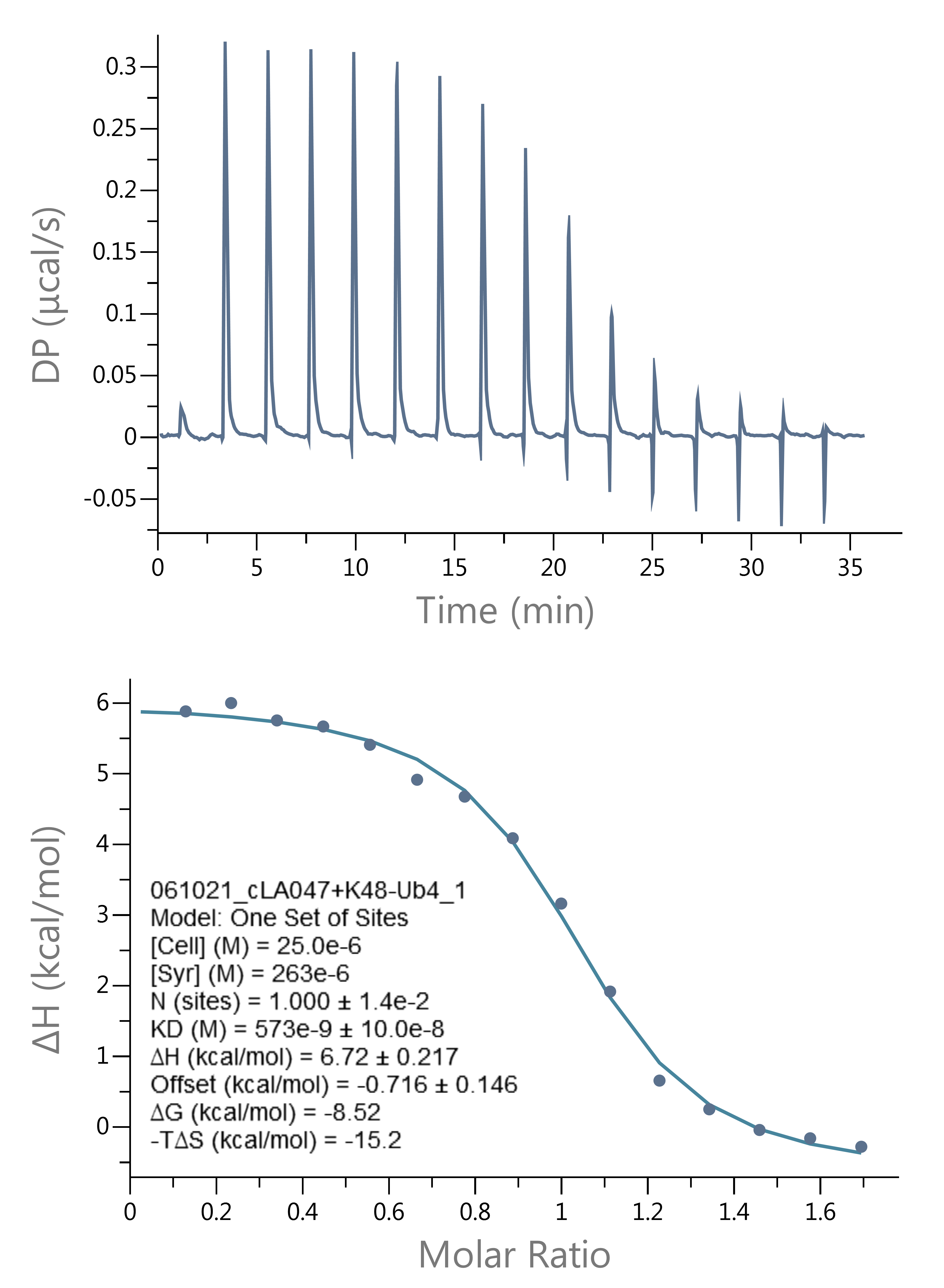

Supplement: Supplementary file 3 — Source data Fig. 1 [file 44319_2026_825_MOESM3_ESM.zip › Figure 1/1D/Ub4_raw_trace.bmp]

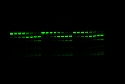

Supplement: Supplementary file 3 — Source data Fig. 1 [file 44319_2026_825_MOESM3_ESM.zip › Figure 1/1B/fluorescent_gel_Ub5/0004307_01_TH.jpg]

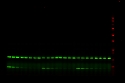

Supplement: Supplementary file 3 — Source data Fig. 1 [file 44319_2026_825_MOESM3_ESM.zip › Figure 1/1B/fluorescent_gel_Ub2/0003874_01_TH.jpg]

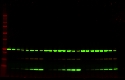

Supplement: Supplementary file 3 — Source data Fig. 1 [file 44319_2026_825_MOESM3_ESM.zip › Figure 1/1B/fluorescent_gel_Ub3/0004355_01_TH.jpg]

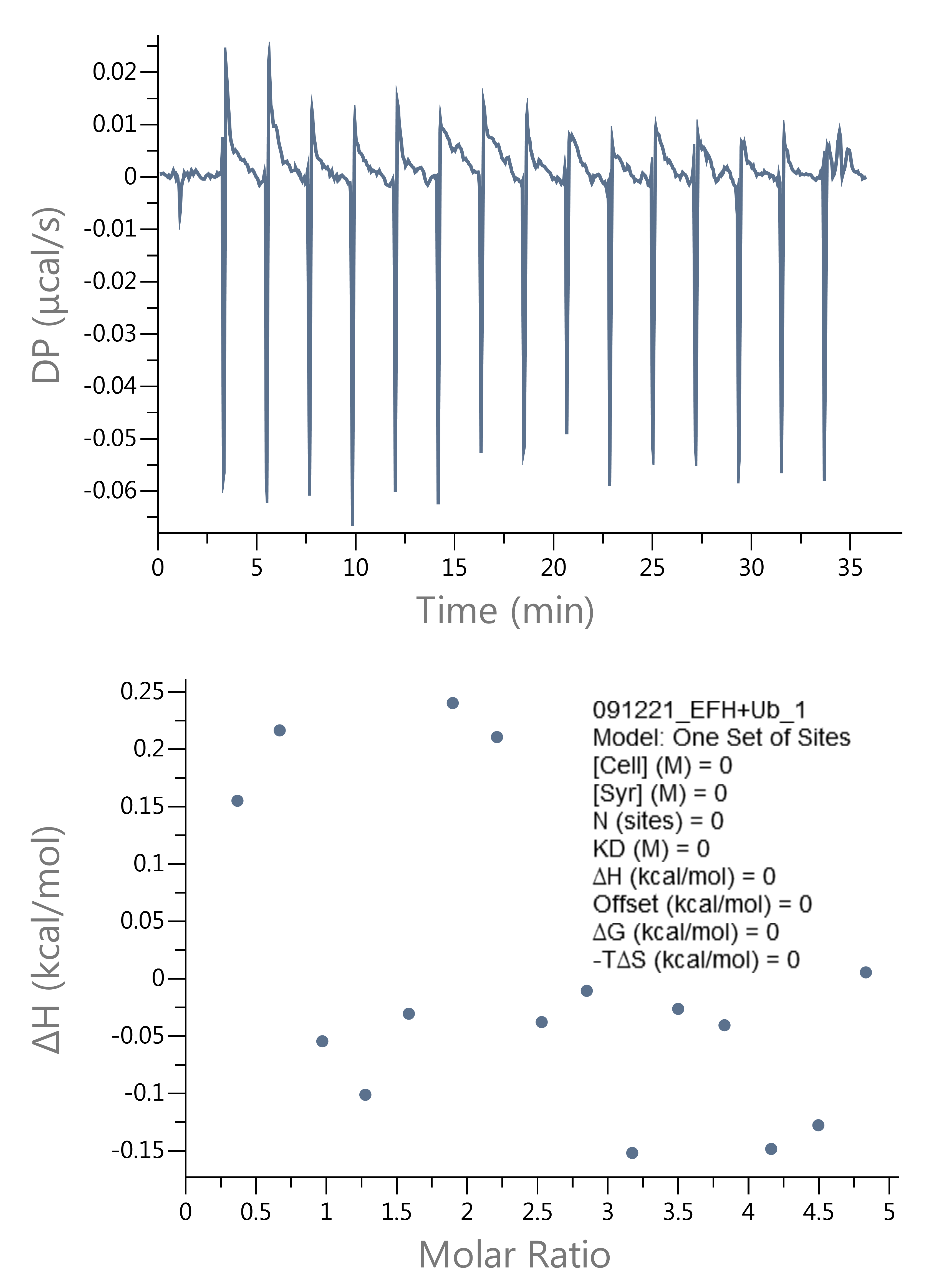

Supplement: Supplementary file 4 — Source data Fig. 2 [file 44319_2026_825_MOESM4_ESM.zip › Figure 2/2F/raw_data.bmp]

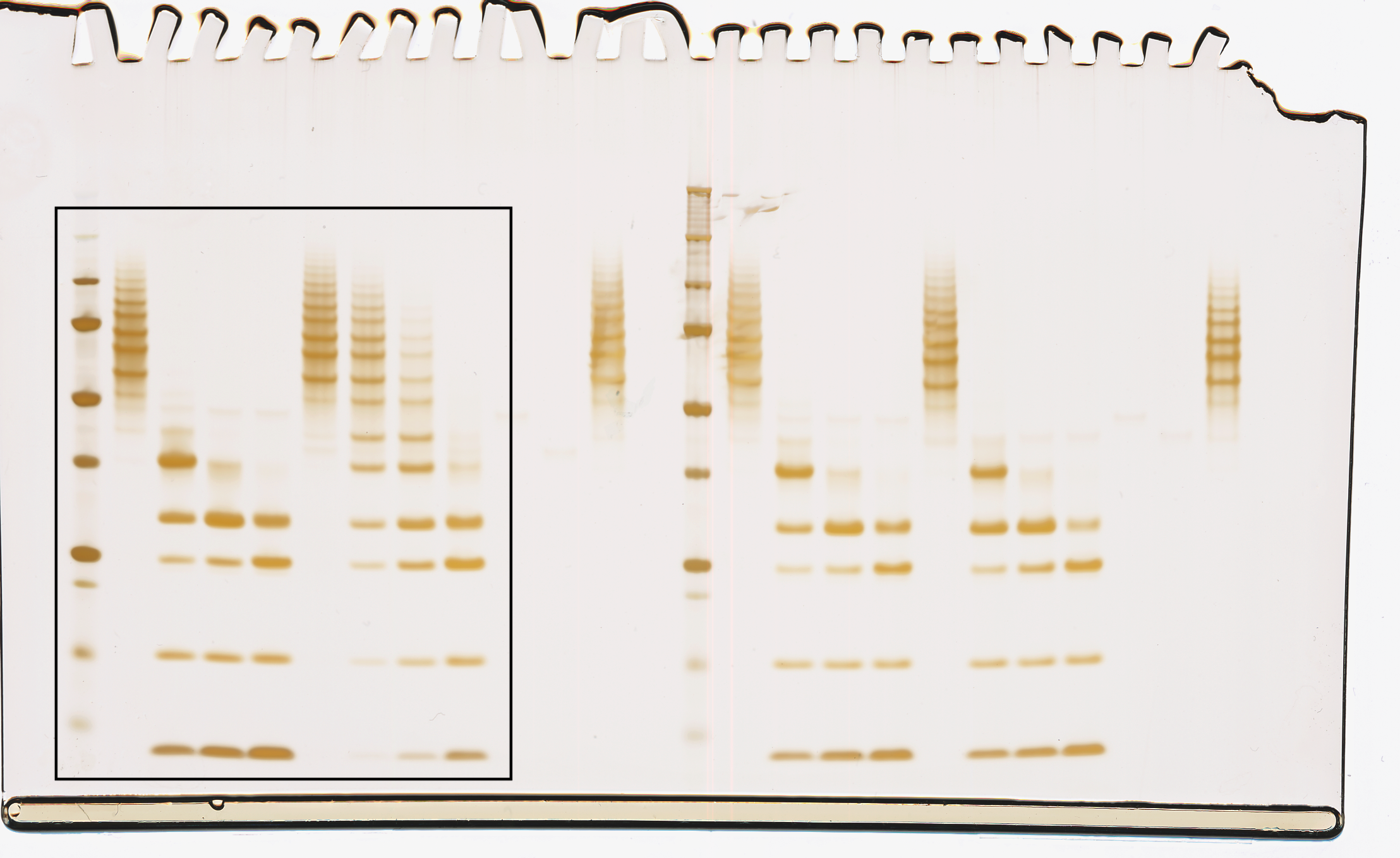

Supplement: Supplementary file 4 — Source data Fig. 2 [file 44319_2026_825_MOESM4_ESM.zip › Figure 2/2C/raw_gel.tif]

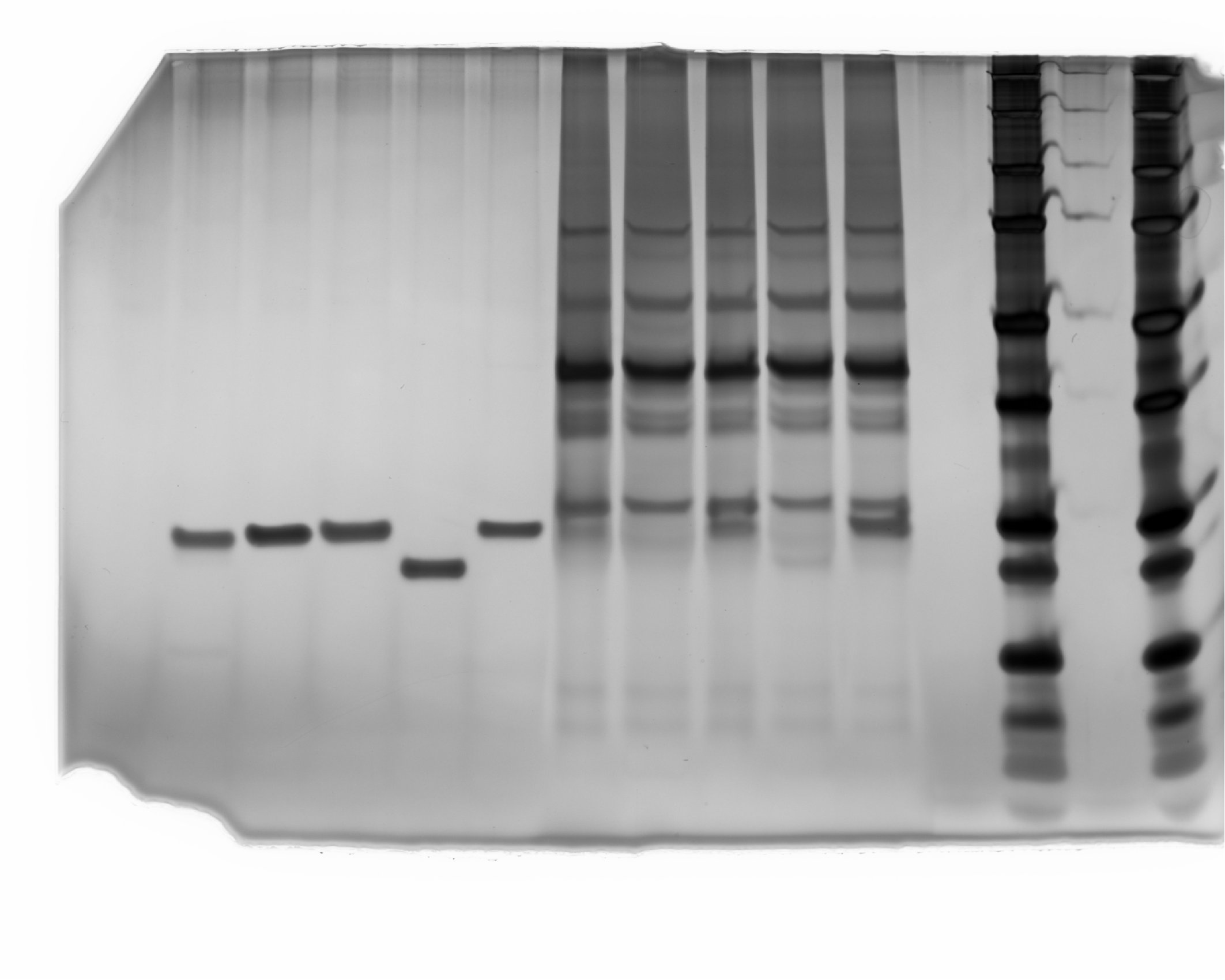

Supplement: Supplementary file 4 — Source data Fig. 2 [file 44319_2026_825_MOESM4_ESM.zip › Figure 2/2E/3E - SDS-PAGE silver stain.tif]

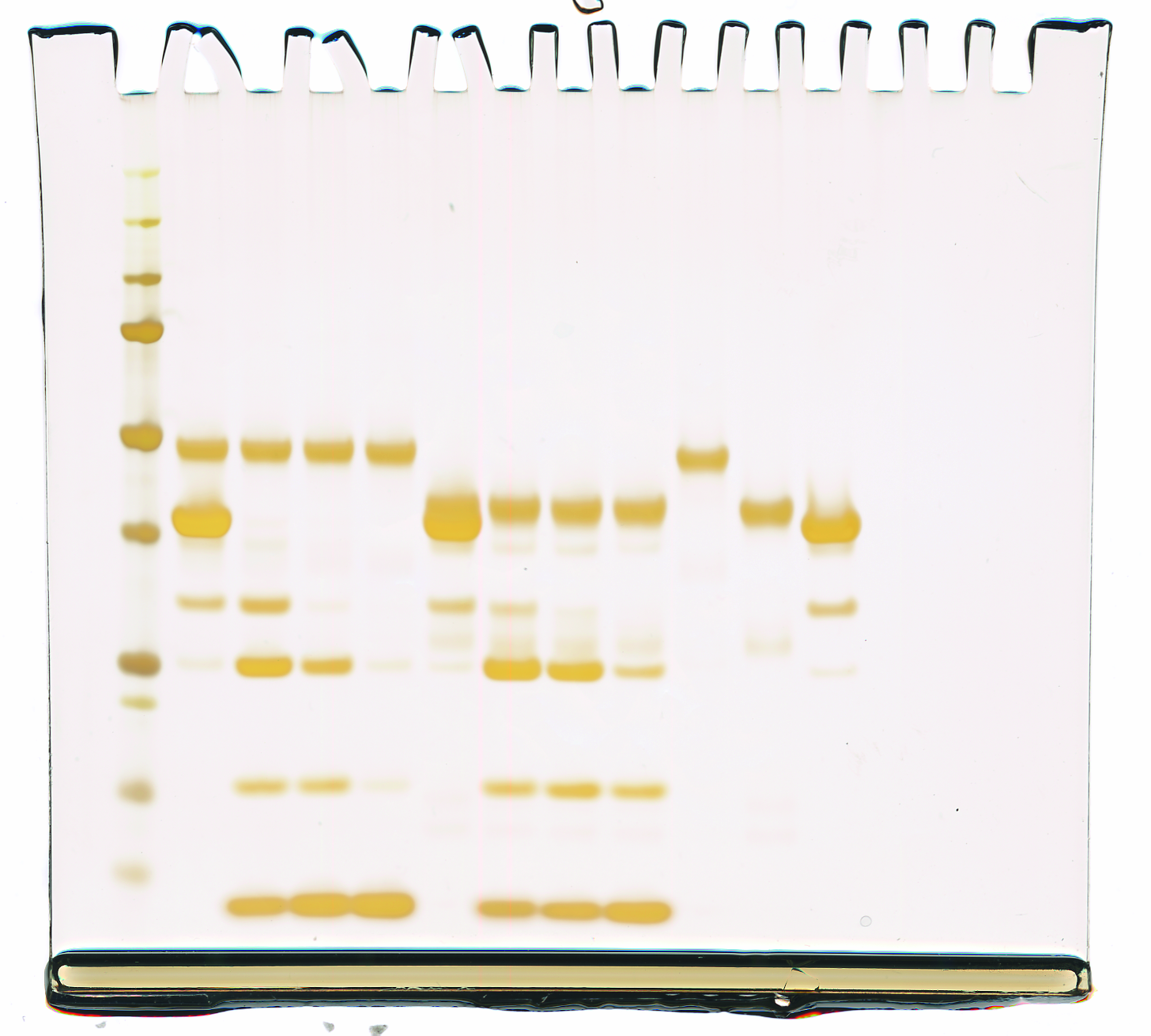

Supplement: Supplementary file 4 — Source data Fig. 2 [file 44319_2026_825_MOESM4_ESM.zip › Figure 2/2B/raw_gel.tif]

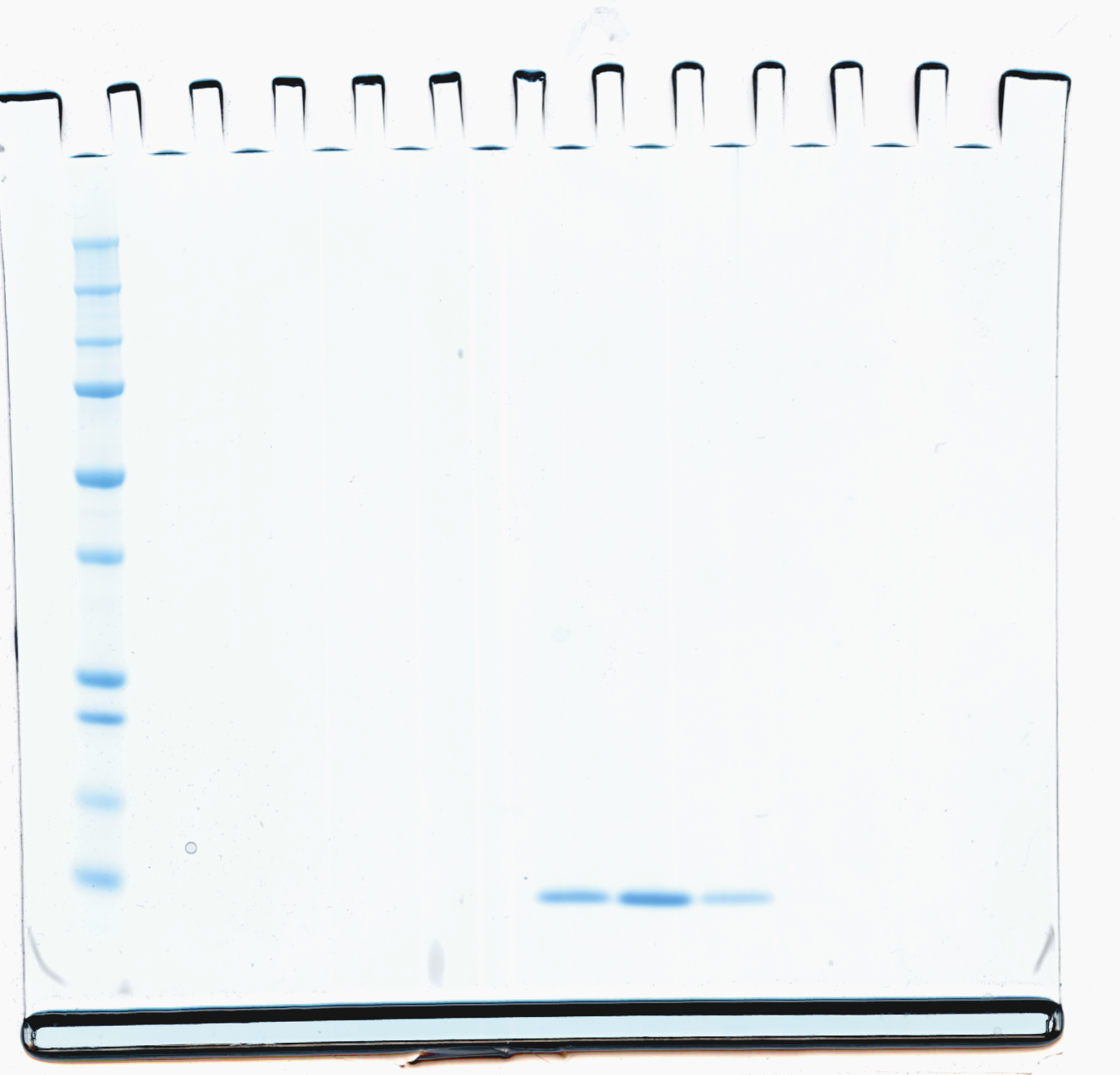

Supplement: Supplementary file 5 — Source data Fig. 3 [file 44319_2026_825_MOESM5_ESM.zip › Figure 3/3E/EFH_raw_gel.tif]

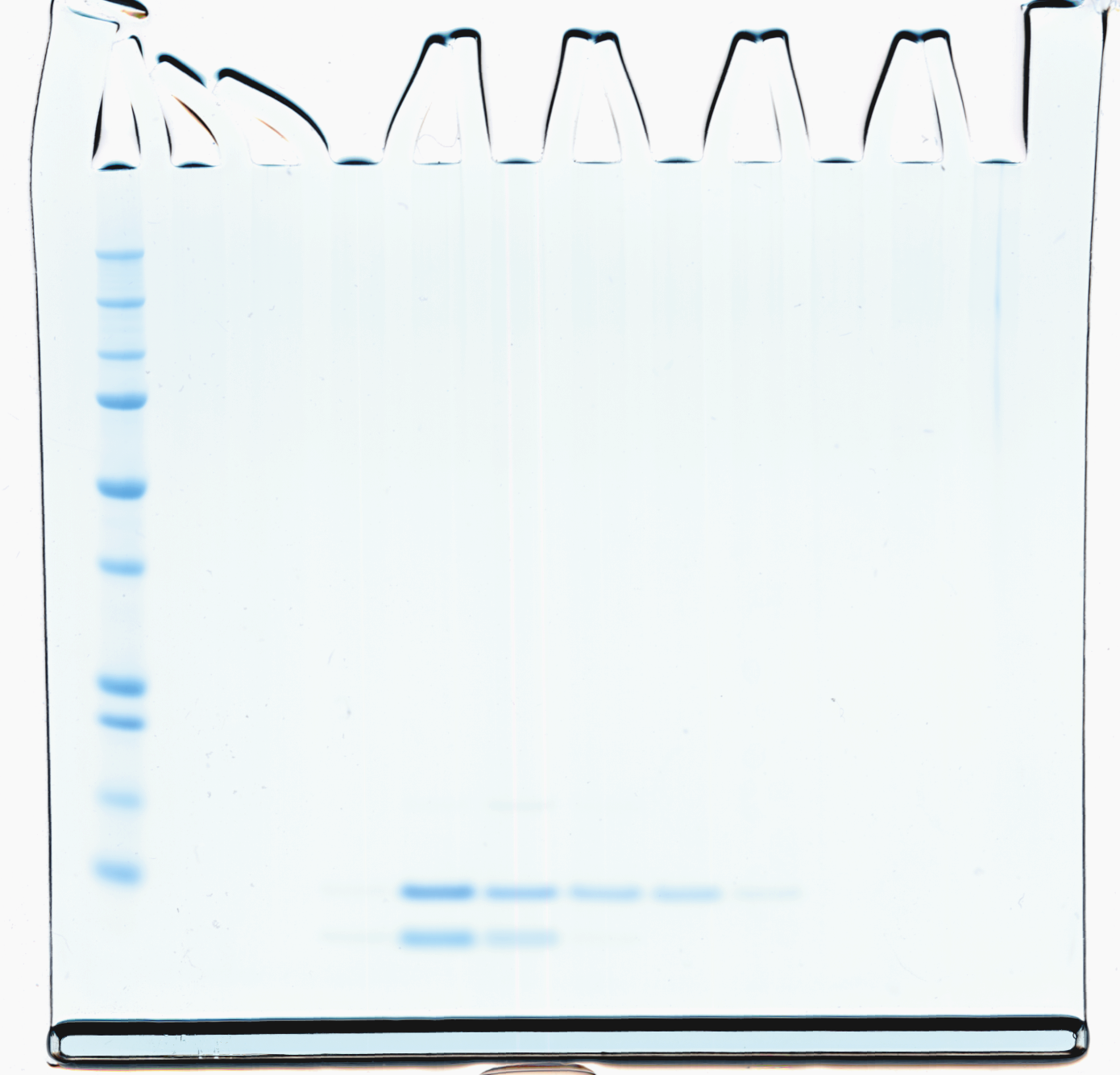

Supplement: Supplementary file 5 — Source data Fig. 3 [file 44319_2026_825_MOESM5_ESM.zip › Figure 3/3E/complex_raw_gel.tif]

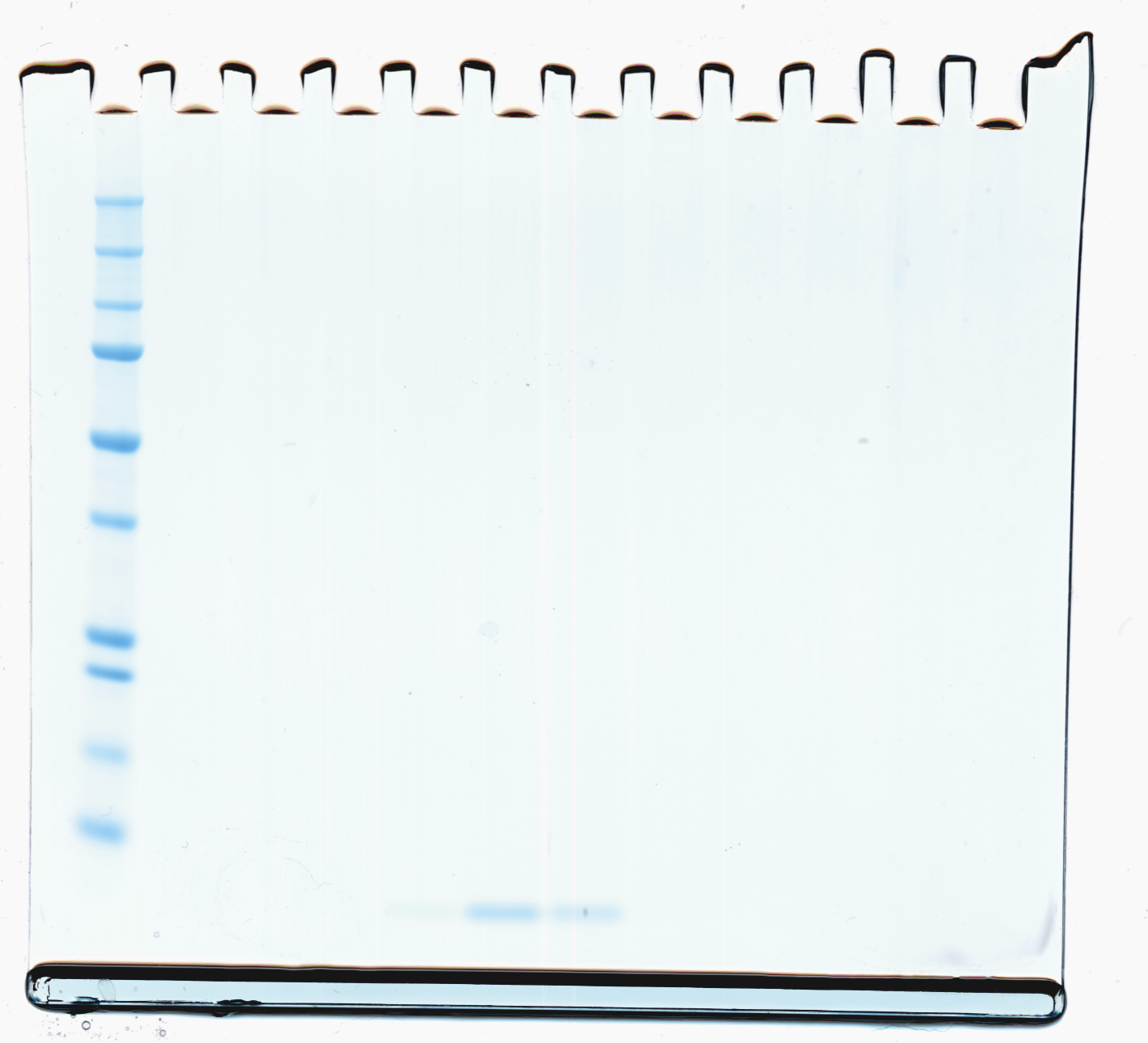

Supplement: Supplementary file 5 — Source data Fig. 3 [file 44319_2026_825_MOESM5_ESM.zip › Figure 3/3E/UBL_raw_gel.tif]

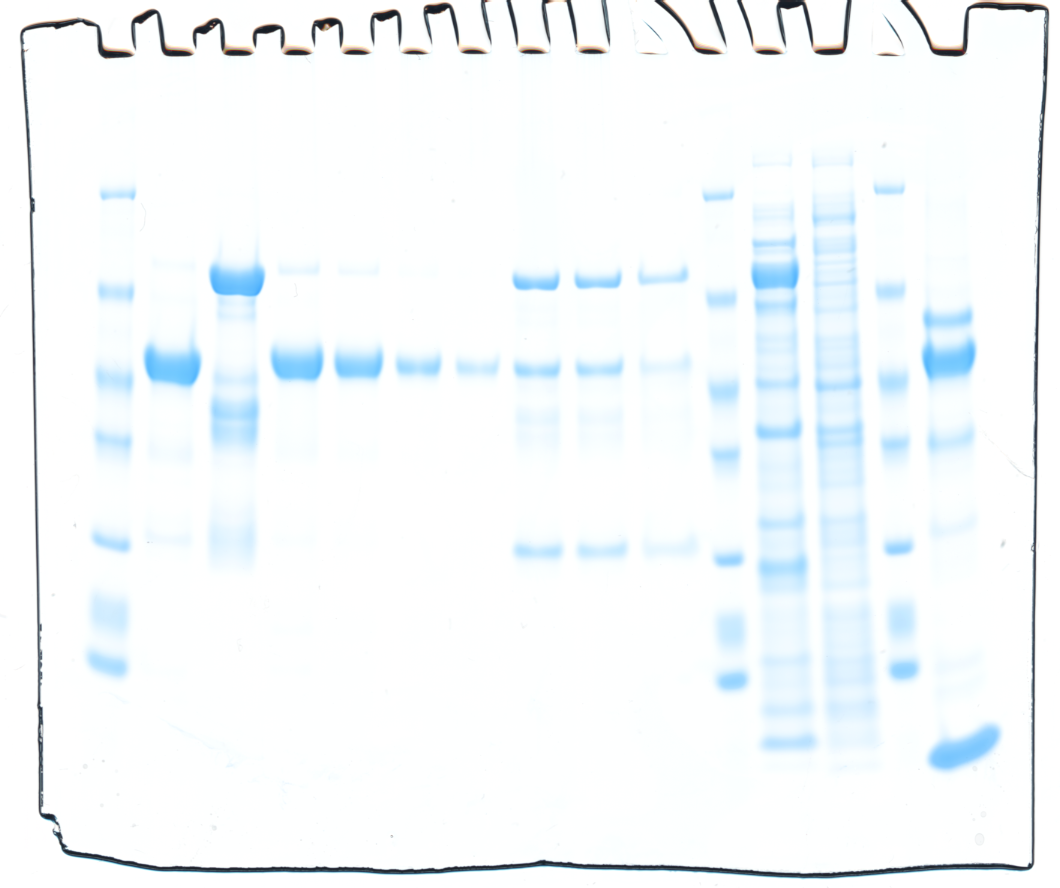

Supplement: Supplementary file 5 — Source data Fig. 3 [file 44319_2026_825_MOESM5_ESM.zip › Figure 3/3B/raw_gel.tiff]

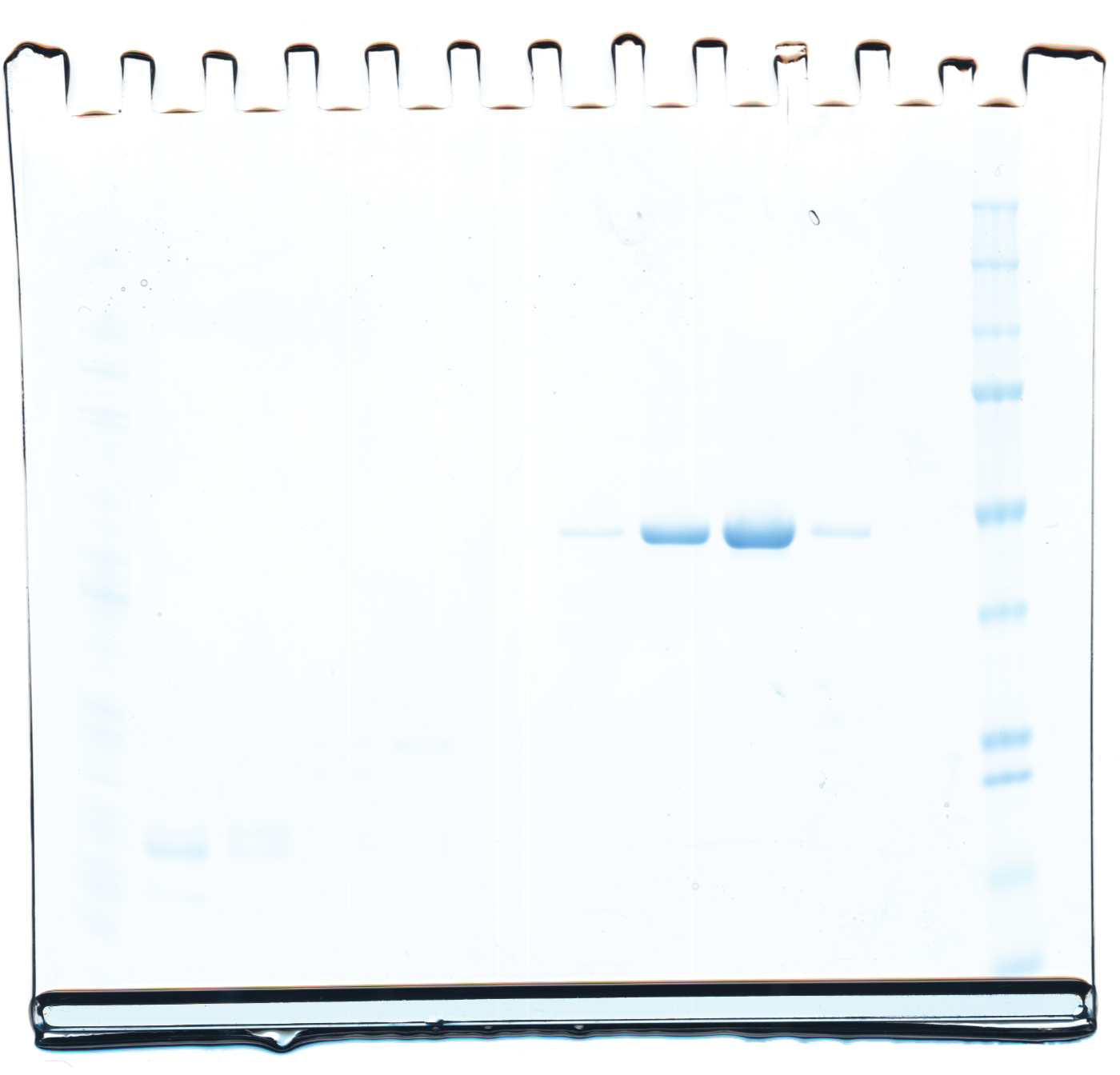

Supplement: Supplementary file 5 — Source data Fig. 3 [file 44319_2026_825_MOESM5_ESM.zip › Figure 3/3C/RAD23A_raw_gel.tif]

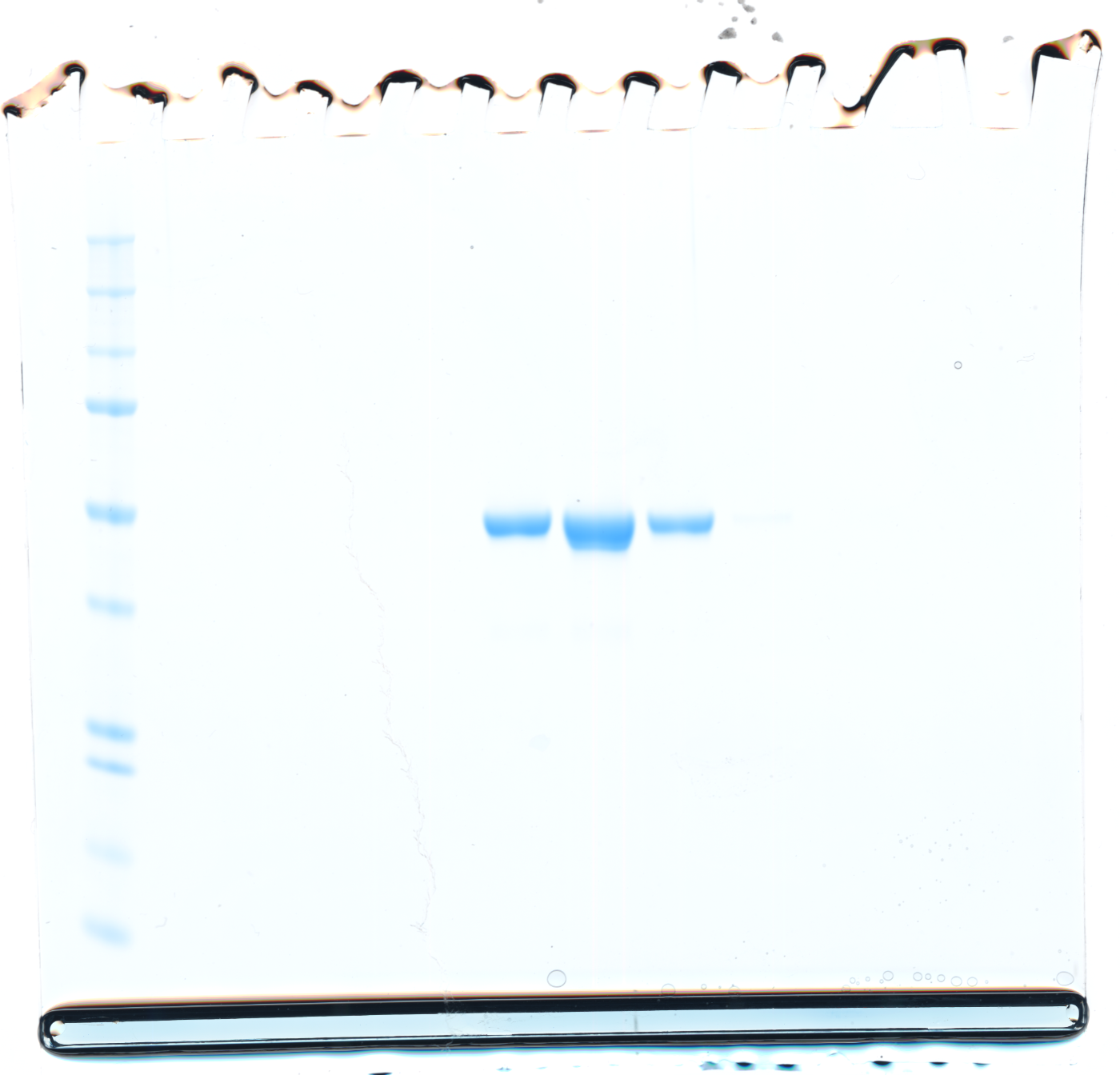

Supplement: Supplementary file 5 — Source data Fig. 3 [file 44319_2026_825_MOESM5_ESM.zip › Figure 3/3C/C51A_raw_gel.tiff]

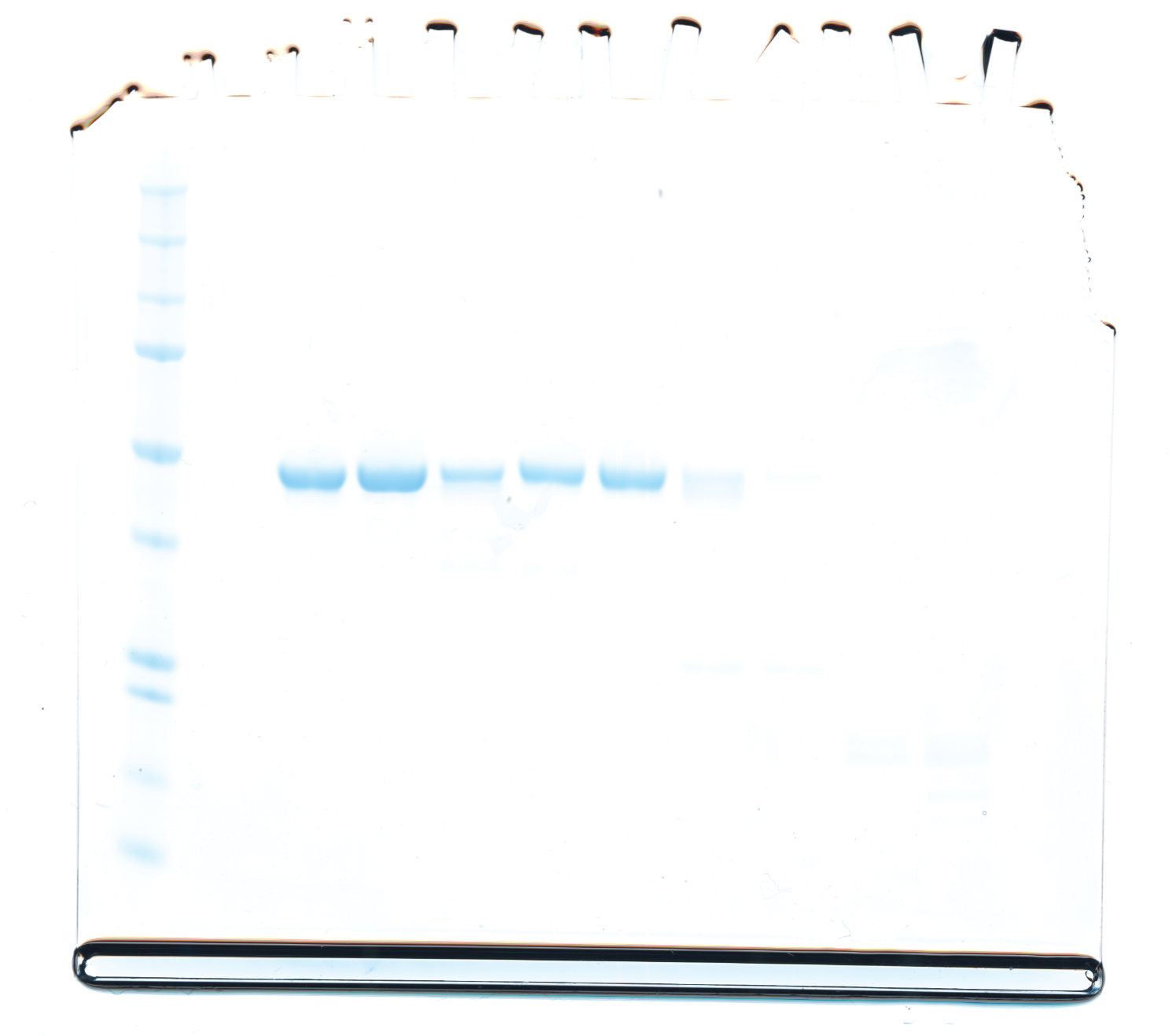

Supplement: Supplementary file 5 — Source data Fig. 3 [file 44319_2026_825_MOESM5_ESM.zip › Figure 3/3C/complex_raw_gel.tif]

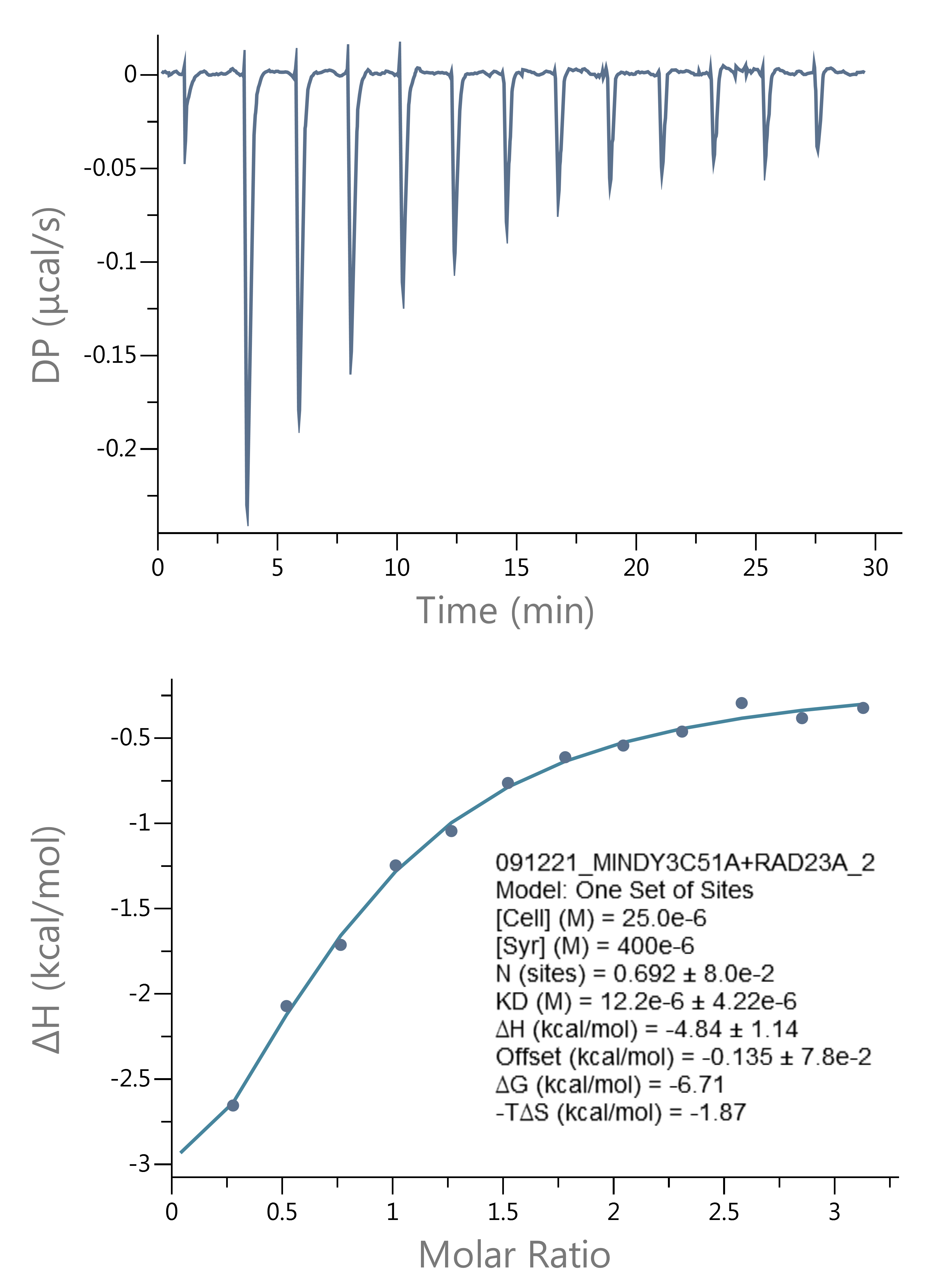

Supplement: Supplementary file 5 — Source data Fig. 3 [file 44319_2026_825_MOESM5_ESM.zip › Figure 3/3D/raw_trace.bmp]

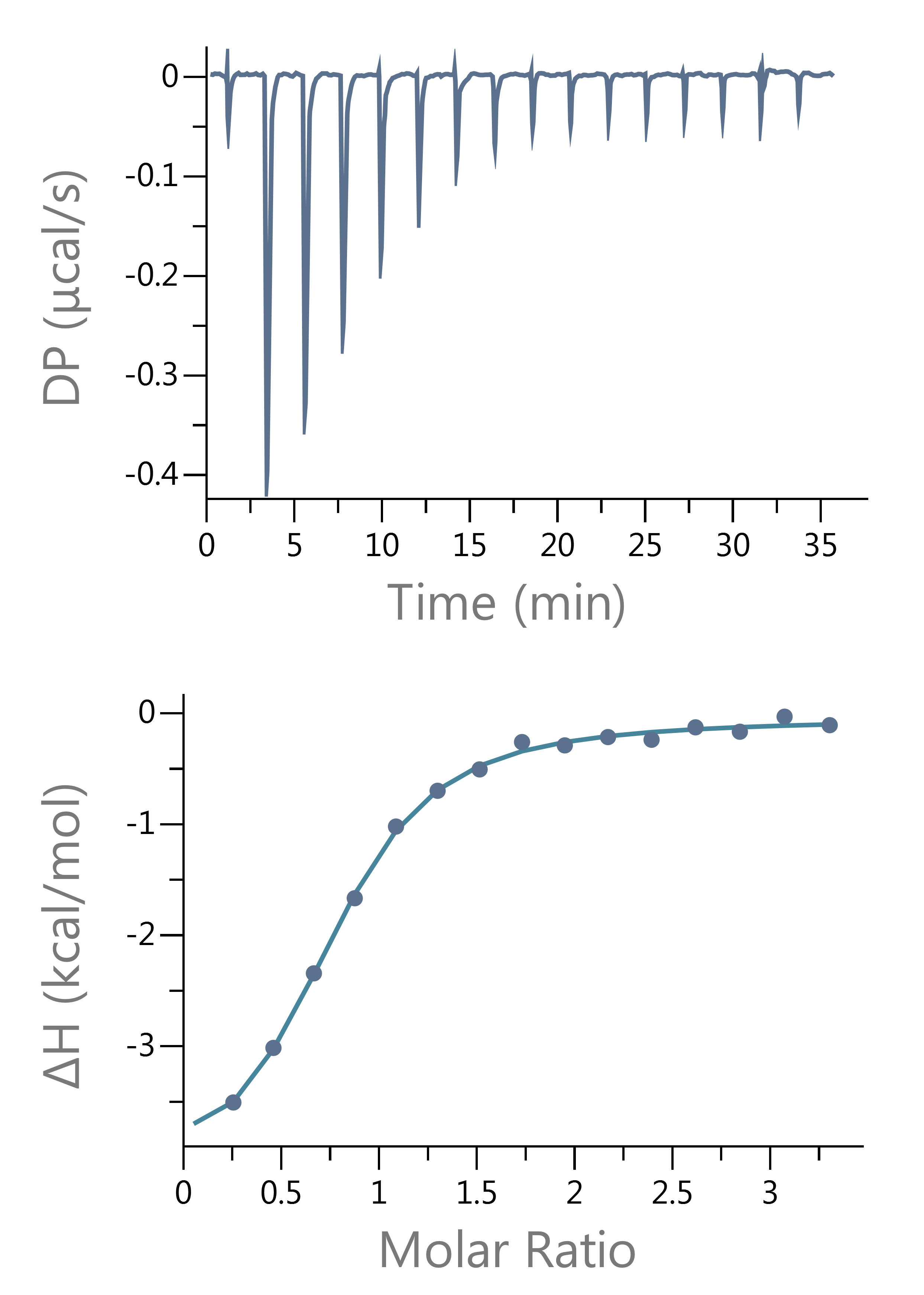

Supplement: Supplementary file 5 — Source data Fig. 3 [file 44319_2026_825_MOESM5_ESM.zip › Figure 3/3F/raw_trace.bmp]

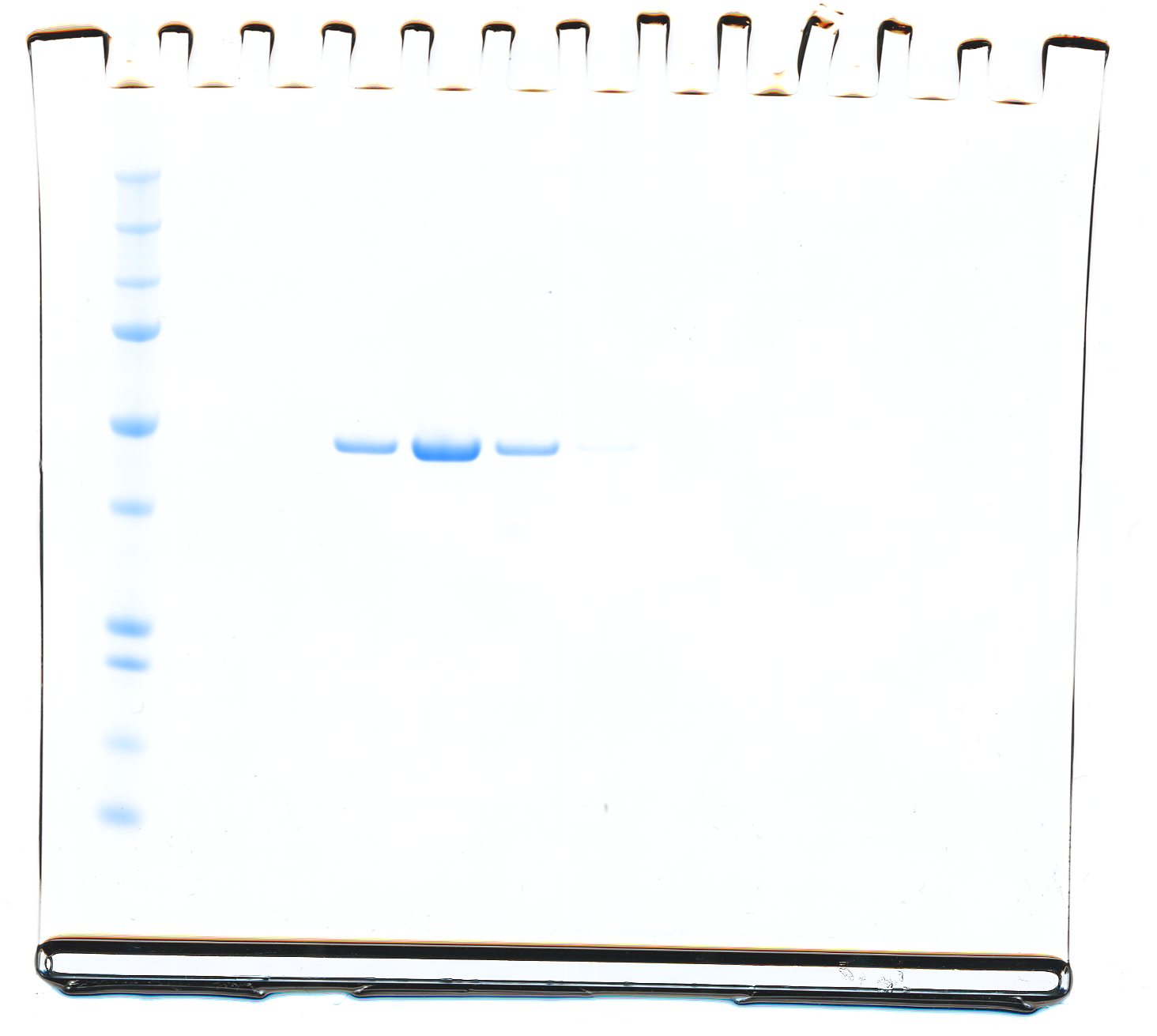

Supplement: Supplementary file 5 — Source data Fig. 3 [file 44319_2026_825_MOESM5_ESM.zip › Figure 3/3G/RAD23A_raw_gel.tif]

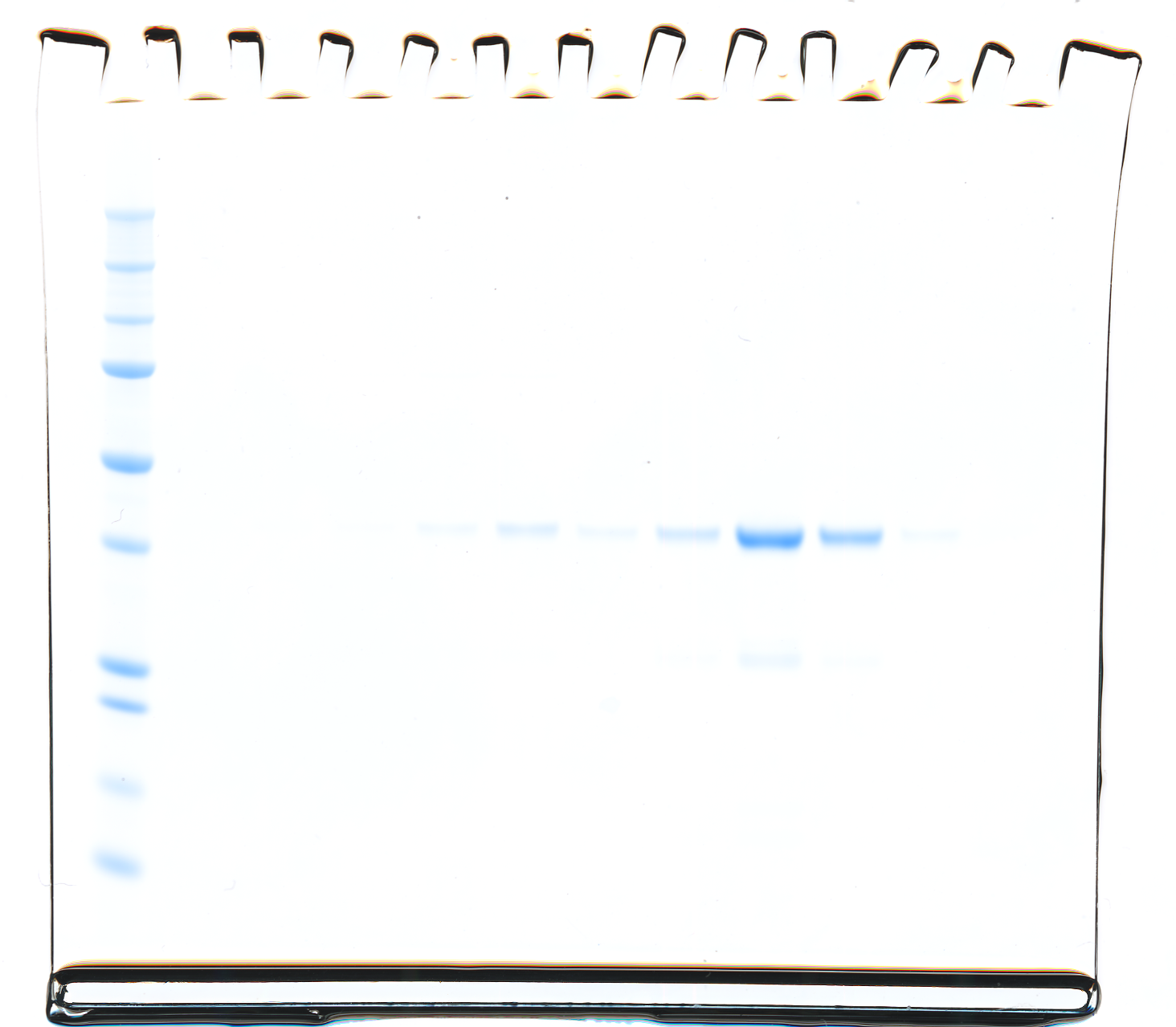

Supplement: Supplementary file 5 — Source data Fig. 3 [file 44319_2026_825_MOESM5_ESM.zip › Figure 3/3G/MINDY3deltaEFH_raw_gel.tif]

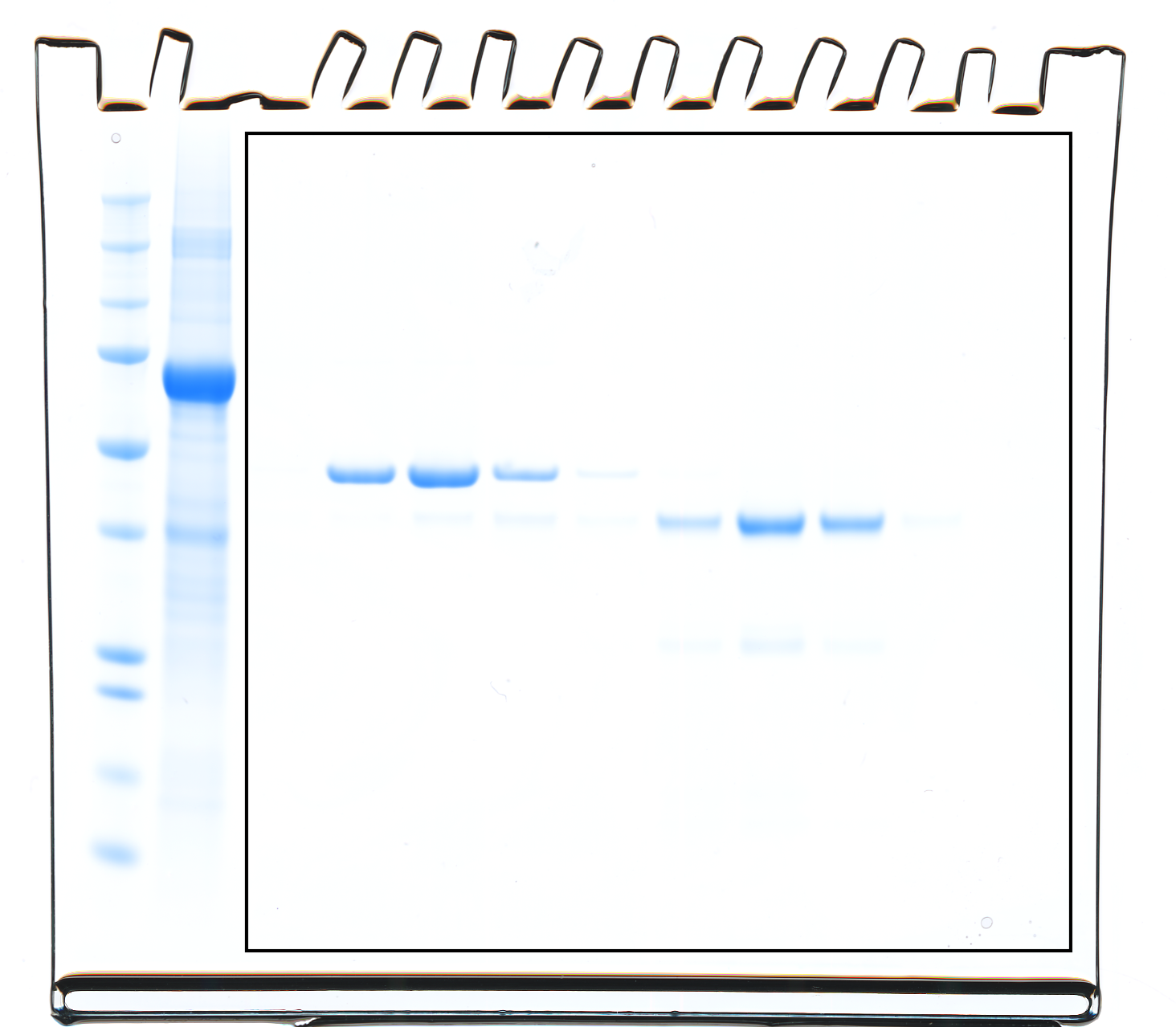

Supplement: Supplementary file 5 — Source data Fig. 3 [file 44319_2026_825_MOESM5_ESM.zip › Figure 3/3G/complex_raw_gel.tif]

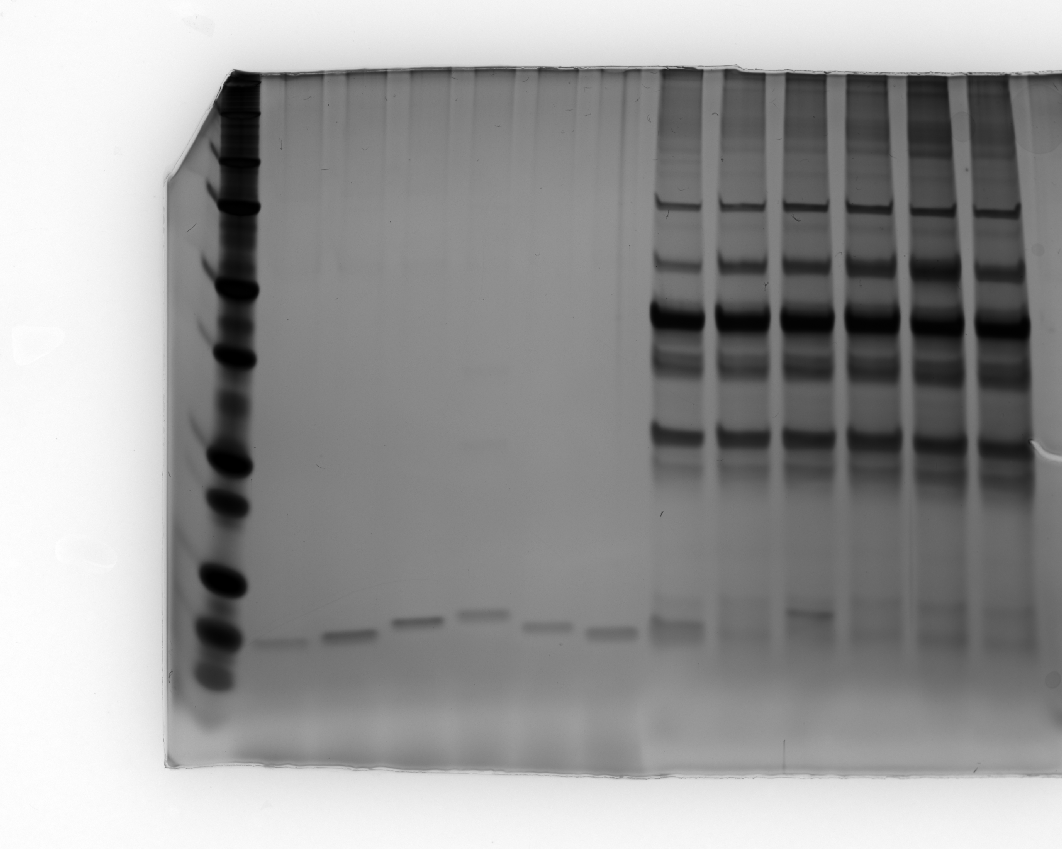

Supplement: Supplementary file 6 — Source data Fig. 4 [file 44319_2026_825_MOESM6_ESM.zip › 4C/4C - SDS-PAGE - silver stain.tif]

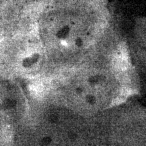

Supplement: Supplementary file 6 — Source data Fig. 4 [file 44319_2026_825_MOESM6_ESM.zip › 4D/Cropped tifs shown in figure/Figure 4D 20230614 GFP-MINDY3 1657 1886 blur 0.5 dam crop.tif]

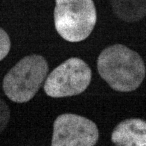

Supplement: Supplementary file 6 — Source data Fig. 4 [file 44319_2026_825_MOESM6_ESM.zip › 4D/Cropped tifs shown in figure/Figure 4D 20230614 GFP-DDB2 1657 2300 blur 0.5 undam cropped.tif]

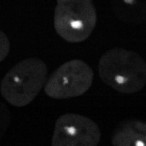

Supplement: Supplementary file 6 — Source data Fig. 4 [file 44319_2026_825_MOESM6_ESM.zip › 4D/Cropped tifs shown in figure/Figure 4D 20230614 GFP-DDB2 1657 2300 blur 0.5 dam cropped.tif]

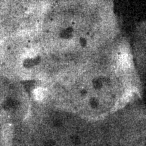

Supplement: Supplementary file 6 — Source data Fig. 4 [file 44319_2026_825_MOESM6_ESM.zip › 4D/Cropped tifs shown in figure/Figure 4D 20230614 GFP-MINDY3 1657 1886 blur 0.5 undam crop.tif]

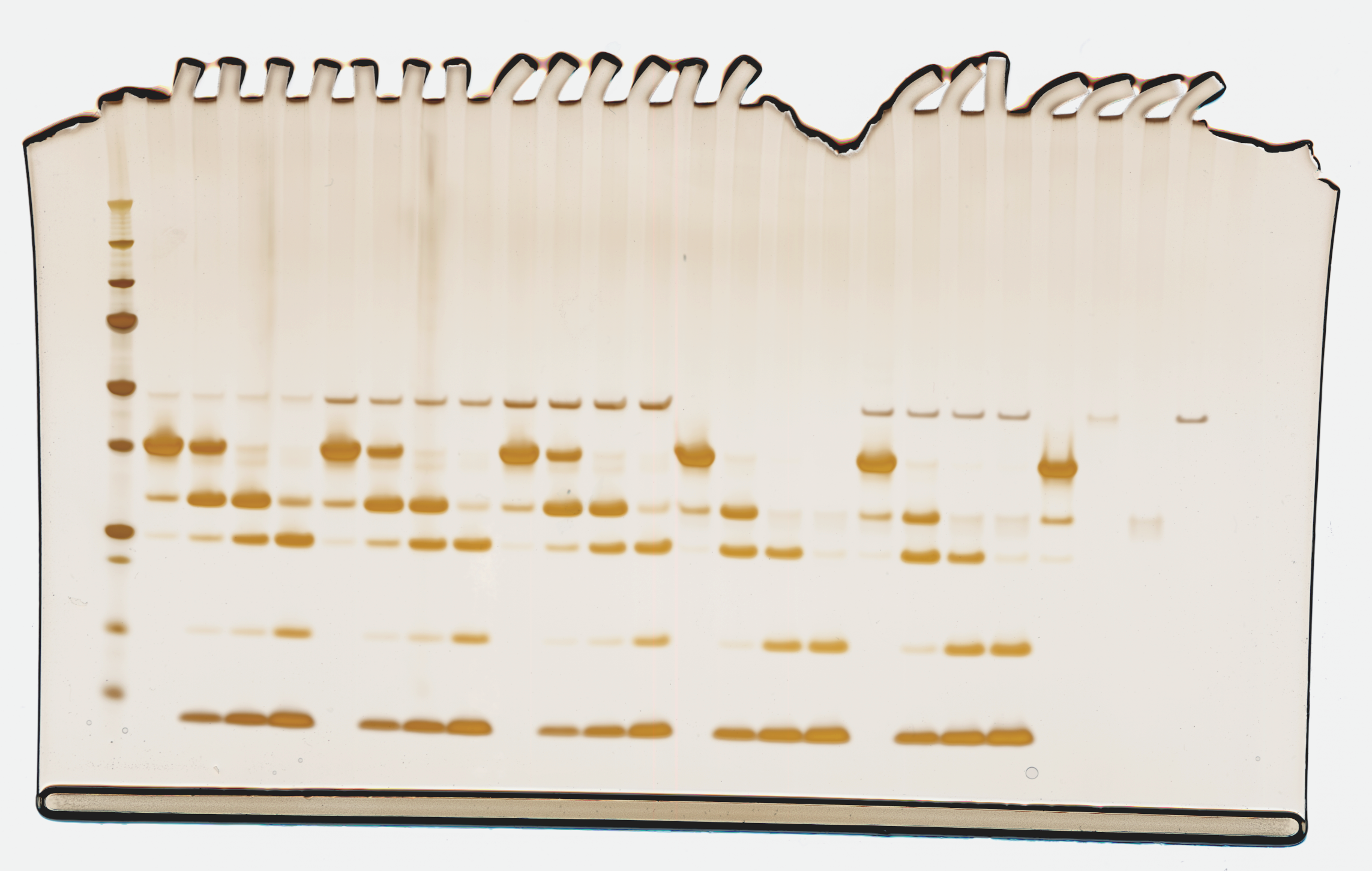

Supplement: Supplementary file 7 — Source data Fig. 5 [file 44319_2026_825_MOESM7_ESM.zip › Figure 5/5A/raw_gel.tif]

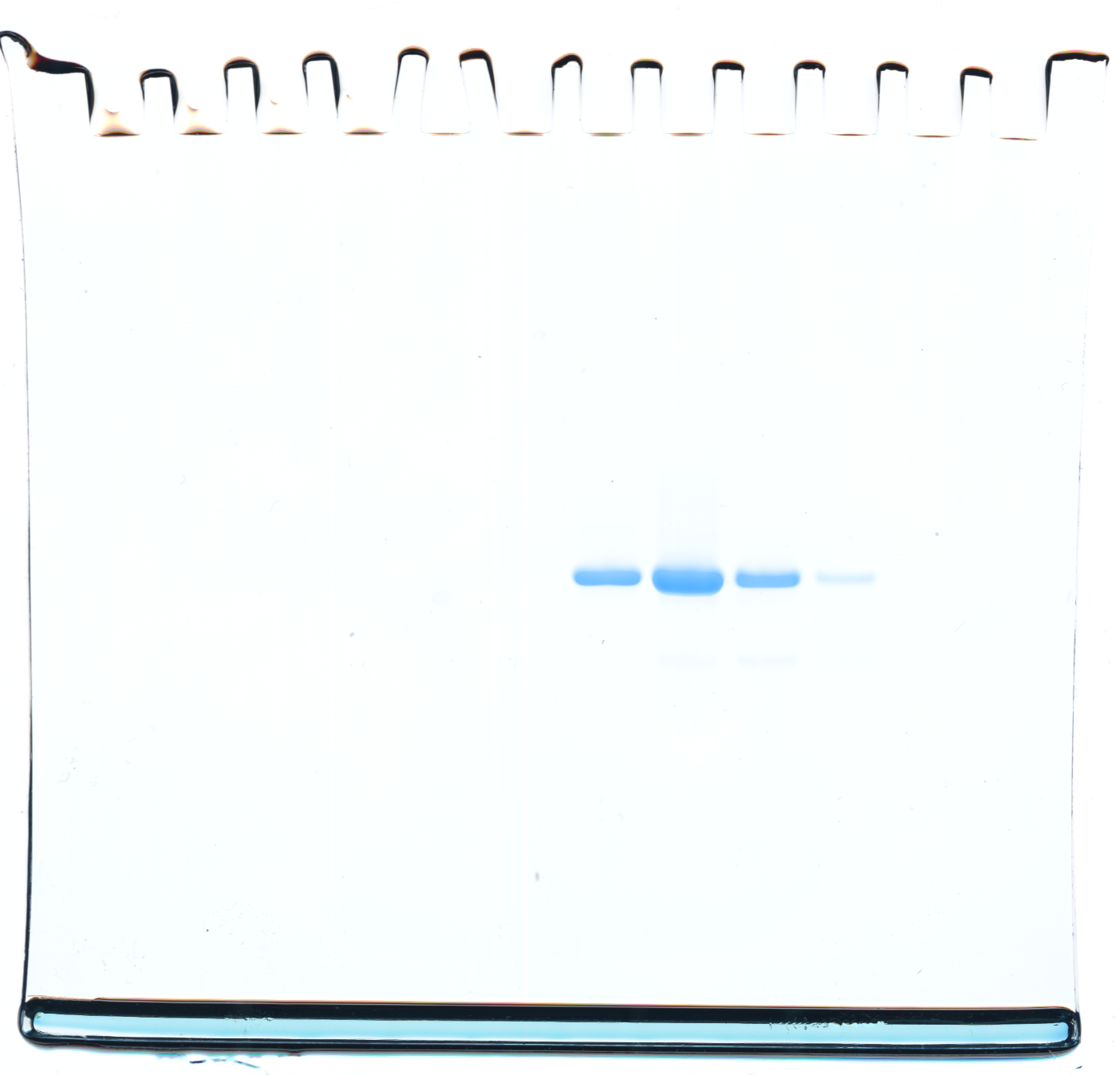

Supplement: Supplementary file 7 — Source data Fig. 5 [file 44319_2026_825_MOESM7_ESM.zip › Figure 5/5C/Ub5_raw_gel.tiff]

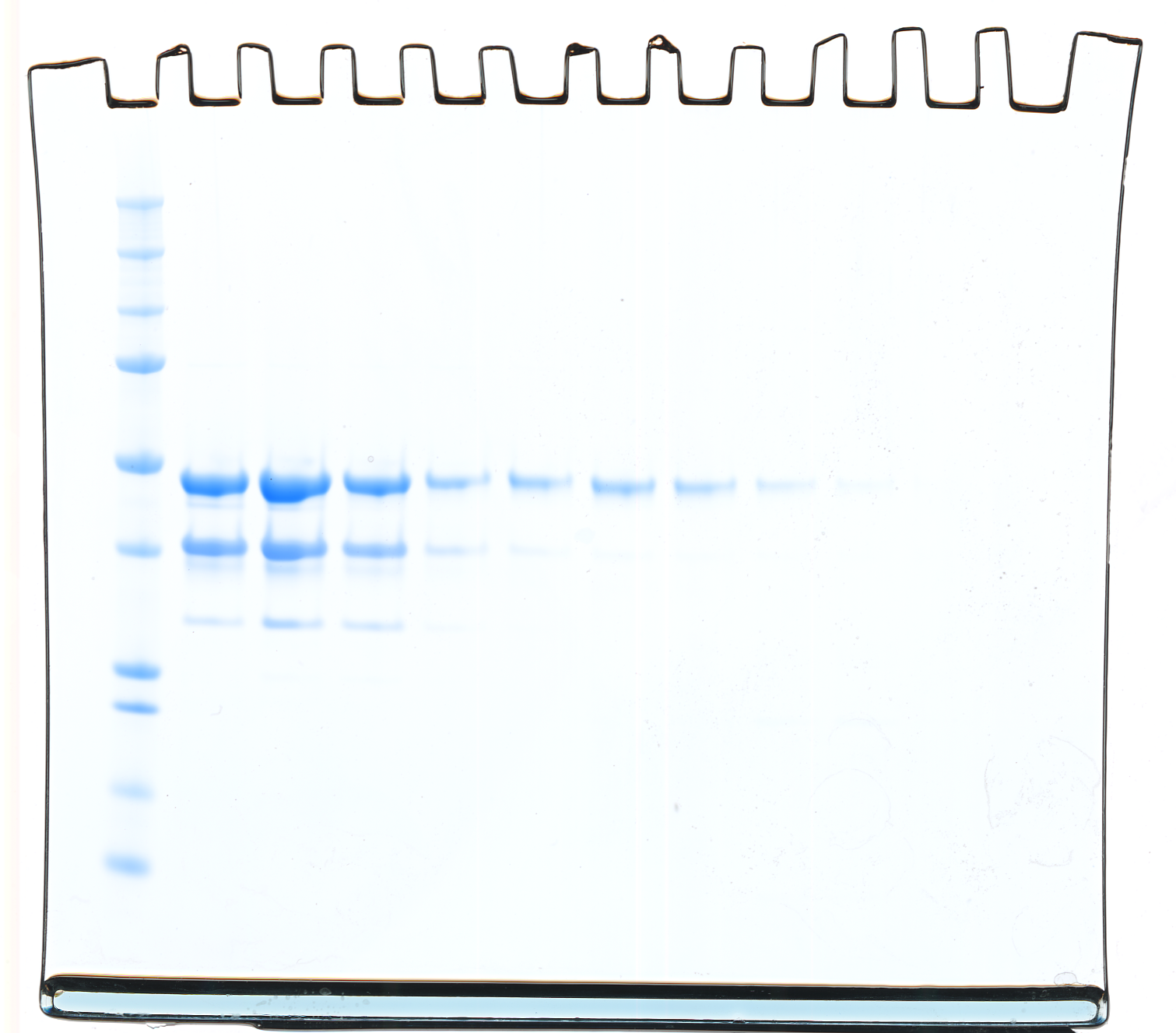

Supplement: Supplementary file 7 — Source data Fig. 5 [file 44319_2026_825_MOESM7_ESM.zip › Figure 5/5C/Complex_raw_gel.tif]

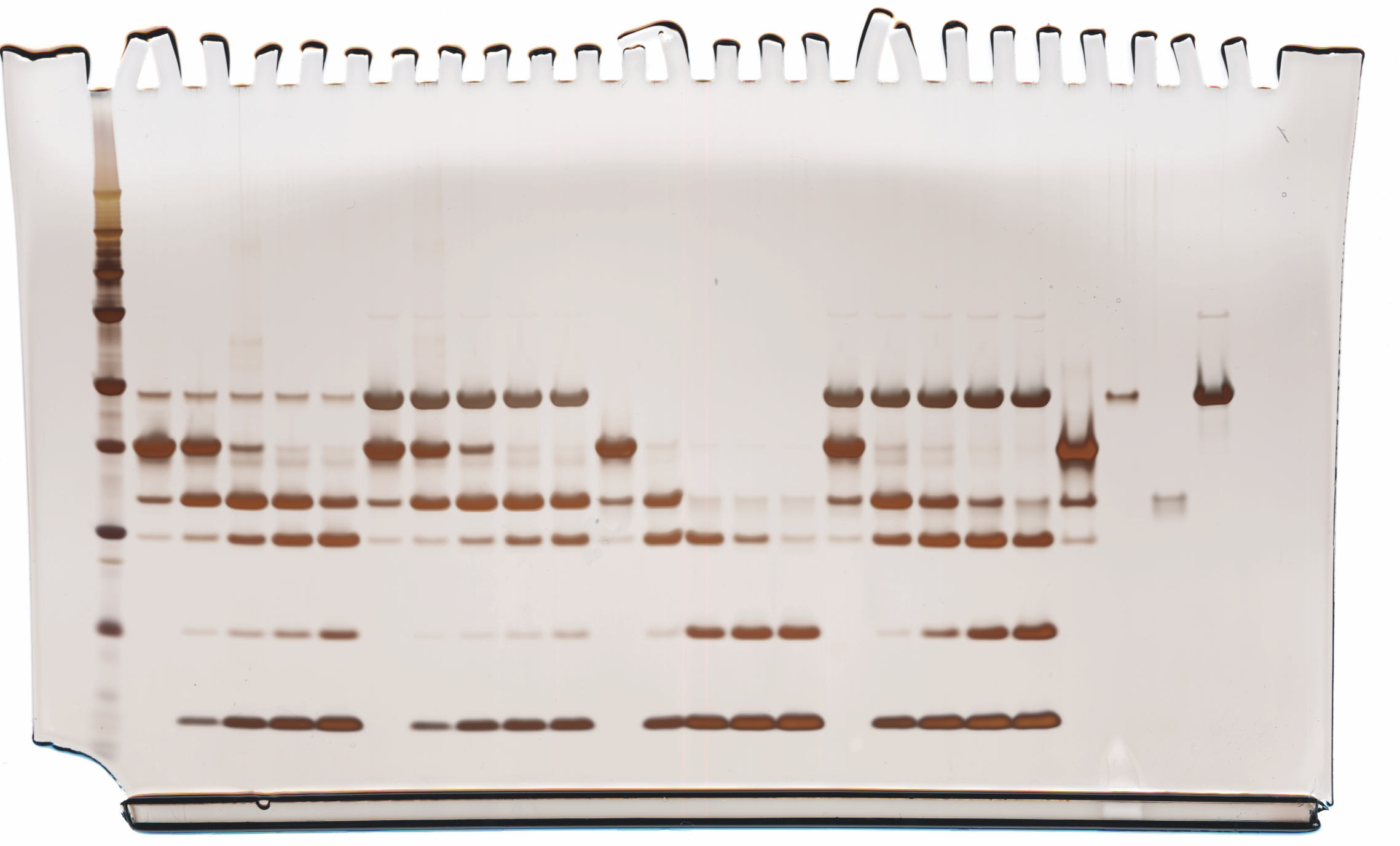

Supplement: Supplementary file 7 — Source data Fig. 5 [file 44319_2026_825_MOESM7_ESM.zip › Figure 5/5B/raw_gel.tif]
