# Supplementary material for: The EF-hand domain of MINDY3 is a ubiquitin and RAD23 UBL-binding domain
Source: EMBO Rep. 2026 Jun 9;27(13):3604–31. doi: 10.1038/s44319-026-00825-1 (PMC13354579; doi:10.1038/s44319-026-00825-1)
Supplement: Supplementary file 8 — Expanded View Figures [file 44319_2026_825_MOESM8_ESM.pdf]

Expanded View Figures

Figure EV1. MINDY3 prefers binding to and cleaving long K48-linked polyUb.

(**A**) Analytical SEC of MINDY3^{C51A} with K48-linked Ub2-5 after pre-incubation. The SEC traces and SDS-PAGE gels for MINDY3^{C51A} are identical across all conditions and shown repeatedly for comparison. (**B**) DUB assay of MINDY3 against K48-linked Ub6. (**C**) DUB assay of MINDY3 against K48-linked polyUb of length >Ub6. (**D**) DUB assay of MINDY3 against K48-linked polyUb of length Ub6+ labelled at the extreme distal moiety with a fluorophore. Miy2 and OTUB1 are endo-DUB controls. (**E**) Superposition of the crystal structure of MINDY3 with the crystal structure of the catalytic domain of MINDY2 (PDB: 6Z49). Source data are available online for this figure.

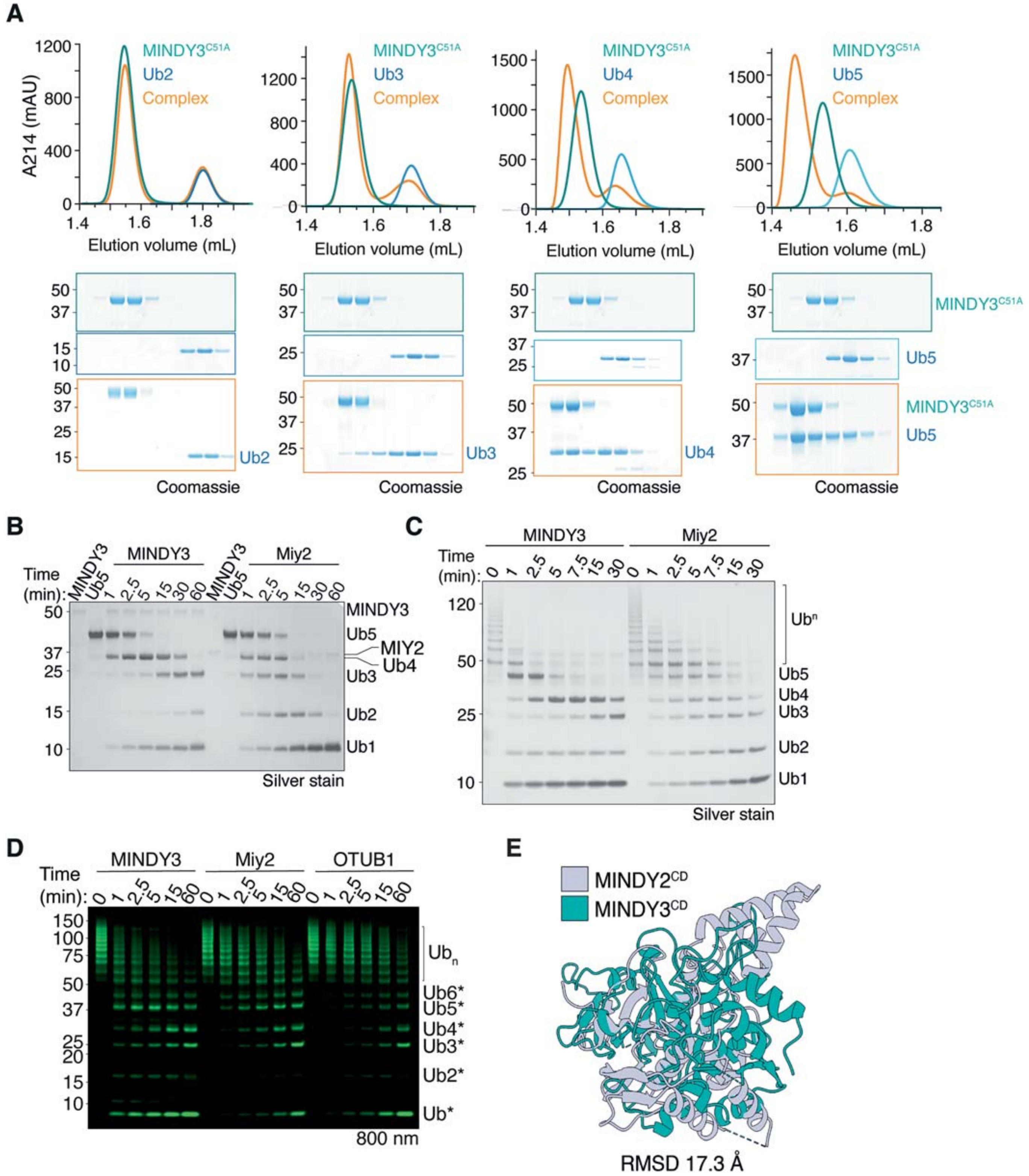
A
MINDY3C51A
Ub2
Complex
A214 (mAU)
Elution volume (mL)
Ub3
Ub4
Ub5
Coomassie
B
Time (min):
MINDY3
Miy2
Ub5
MIY2
Ub4
Ub3
Ub2
Ub1
Silver stain
C
Ubn
D
OTUB1
Ub6*
Ub5*
Ub4*
Ub3*
Ub2*
Ub*
800 nm
E
MINDY2CD
MINDY3CD
RMSD 17.3 Å

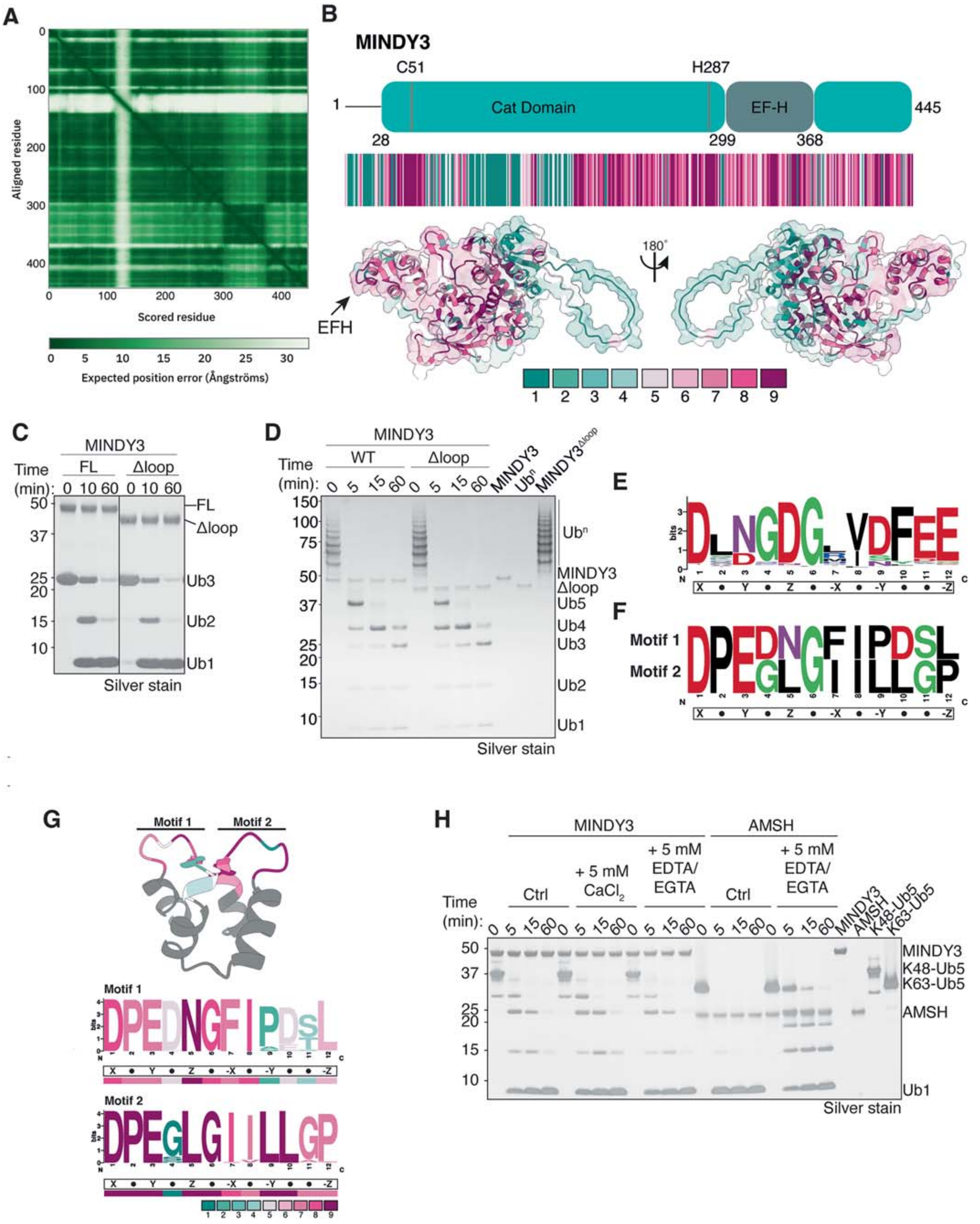
A
Aligned residue
Scored residue
Expected position error (Ångströms)
B
MINDY3
C51
H287
Cat Domain
EF-H
1
445
28
299
368
180°
EFH
C
MINDY3
Time (min):
FL
Δloop
FL
Δloop
Ub3
Ub2
Ub1
Silver stain
D
MINDY3
WT
Δloop
Time (min):
MINDY3
Ub^n
MINDY3^Δloop
Ub^n
MINDY3
Δloop
Ub5
Ub4
Ub3
Ub2
Ub1
Silver stain
E
F
Motif 1
Motif 2
G
Motif 1
Motif 2
H
MINDY3
AMSH
Ctrl
+ 5 mM CaCl2
+ 5 mM EDTA/ EGTA
Ctrl
+ 5 mM EDTA/ EGTA
Time (min):
MINDY3
AMSH
K48-Ub5
K63-Ub5
MINDY3
K48-Ub5
K63-Ub5
AMSH
Ub1
Silver stain

◀ **Figure EV2. MINDY3$^{EF\text{-}hand}$ lacks metal-coordinating residues and does not require Ca^{2+} for function.**

(**A**) PAE plot of the AlphaFold prediction of full-length MINDY3. Low confidence regions correlate to the flexible loop region in MINDY3. (**B**) Conservation analysis of each amino acid position of MINDY3 was generated by Consurf (Ashkenazy et al, 2016) from the sequences of 186 vertebrate species. Each residue is given a score between 1 and 9, with 1 being the least conserved and 9 being the most conserved. Consurf analysis is then mapped onto the AlphaFold prediction for FL MINDY3. (**C**) DUB assay of MINDY3 and MINDY3$^{\Delta loop}$ against K48-linked Ub3. (**D**) DUB assay of MINDY3 and MINDY3$^{\Delta loop}$ against K48-linked polyUb of Ub6+. (**E**) WebLogo visualisation of sequence conservation of the EF-hand motif generated from 878 vertebrate genes. The overall height of a letter stack indicates the level of conservation at that position, while the height of an individual letter indicates the relative frequency of the amino acid at that position (Crooks et al, 2004). (**F**) WebLogo visualisation comparing the two EF-hand motifs in MINDY3. The height of an individual letter indicates the relative frequency of the amino acid at the position. (**G**) Upper: MINDY3$^{EF\text{-}hand}$ with the EF-hand motifs coloured according to Consurf analysis of MINDY3 from 186 vertebrate genes. Lower: WebLogo visualisation of sequence conservation of the MINDY3$^{EF\text{-}hand}$ motif generated from 186 vertebrate genes. The overall height of a letter stack indicates the level of conservation at that position, while the height of an individual letter indicates the relative frequency of the amino acid at that position (Crooks et al, 2004). Coloured by Consurf analysis. (**H**) DUB assay in which MINDY3 was untreated or pre-incubated with $CaCl_2$ or EDTA/EGTA prior to incubation with K48-linked Ub5. AMSH, a metalloprotease, was used as a control with K63-linked Ub5.

© The Author(s)

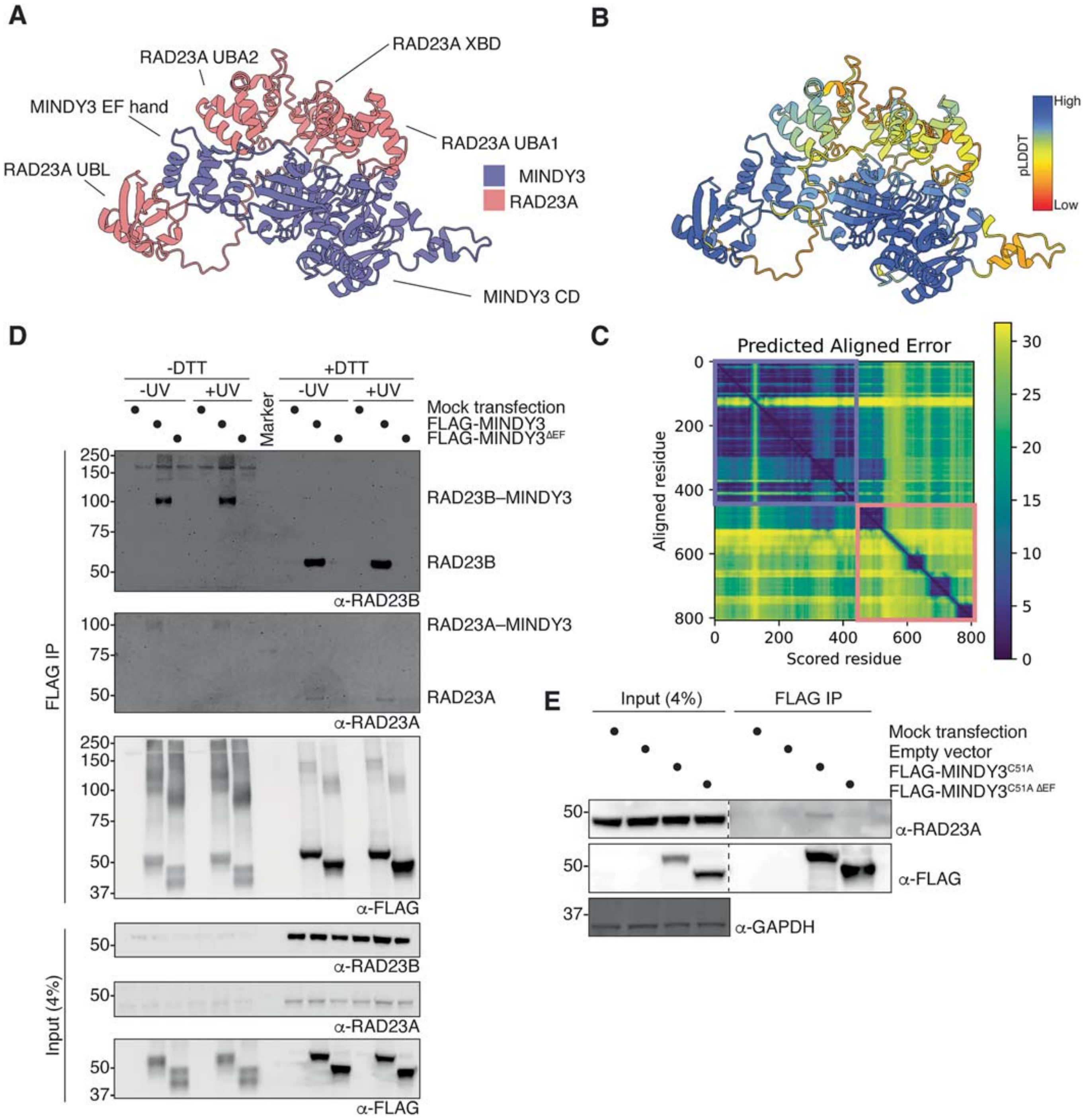

Figure EV3. MINDY3 interacts with RAD23A/B in cells.

(**A**) AlphaFold 2 prediction of full-length MINDY3 with full-length RAD23A. (**B**) Alphafold 2 prediction as in (A), but coloured according to confidence based on pLDDT score. (**C**) Associated PAE plot for AlphaFold prediction. Coloured boxes indicate individual proteins as in Fig. EV4A. (**D**) Crosslinking IP of MINDY3 from HEK293 cells. Cells were transiently transfected with FLAG-MINDY3 ± EF-hand and crosslinked with DSP before FLAG IP. Input and elution fractions (±DTT to resolve crosslinks) were analysed via western blotting with the indicated antibodies. (**E**) Co-immunoprecipitation of MINDY3^{C51A} and RAD23A from HEK293 cells. Cells were transiently transfected with catalytically dead FLAG-MINDY3 ± EF-hand before FLAG IP. Input and elution fractions were analysed via western blotting with the indicated antibodies.

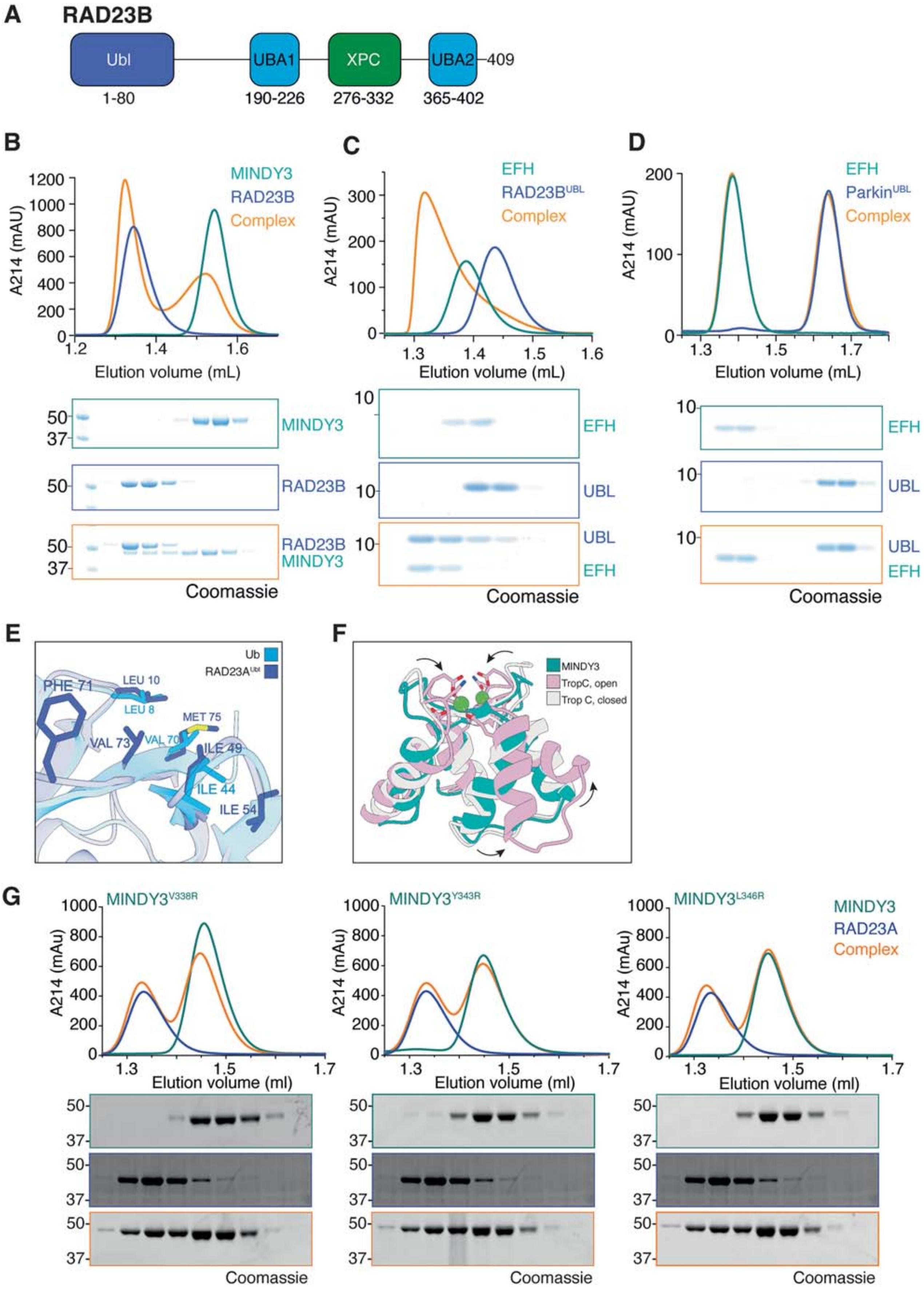
A
RAD23B
Ubl
UBA1
XPC
UBA2
409
1-80
190-226
276-332
365-402
B
MINDY3
RAD23B
Complex
A214 (mAU)
Elution volume (mL)
Coomassie
C
EFH
RAD23B^UBL
Complex
UBL
D
Parkin^UBL
E
Ub
RAD23A^Ubl
PHE 71
LEU 10
LEU 8
MET 75
VAL 73
VAL 70
ILE 49
ILE 44
ILE 54
F
TropC, open
Trop C, closed
G
MINDY3^V338R
MINDY3^Y343R
MINDY3^L346R
RAD23A
Elution volume (ml)

 © The Author(s)

Figure EV4. MINDY3 interacts with RAD23 UBLs via EF-hand Site A.

(**A**) Domain schematic of RAD23B showing the N-terminal UBL domain, the two UBA domains and the XPC-binding domain. (**B**) SEC analysis of MINDY3 and FL RAD23B. (**C**) SEC analysis of MINDY3$^{EF\text{-}hand}$ with RAD23B^{UBL}. (**D**) SEC analysis of MINDY3$^{EF\text{-}hand}$ with Parkin^{UBL}. (**E**) Comparison of the interaction interface of RAD23A^{UBL} with the I44 patch of Ub. (**F**) Transition between open and closed forms of the EF-hand in Troponin C (PDB: 5TNC) superposed with MINDY3$^{EF\text{-}hand}$. (**G**) SEC analyses using point mutants of MINDY3$^{EF\text{-}hand}$ at the EFH-UBL interaction interface. The SDS-PAGE gel panels and SEC traces for RAD23A are identical across the three conditions and are shown repeatedly for comparison.

© The Author(s)

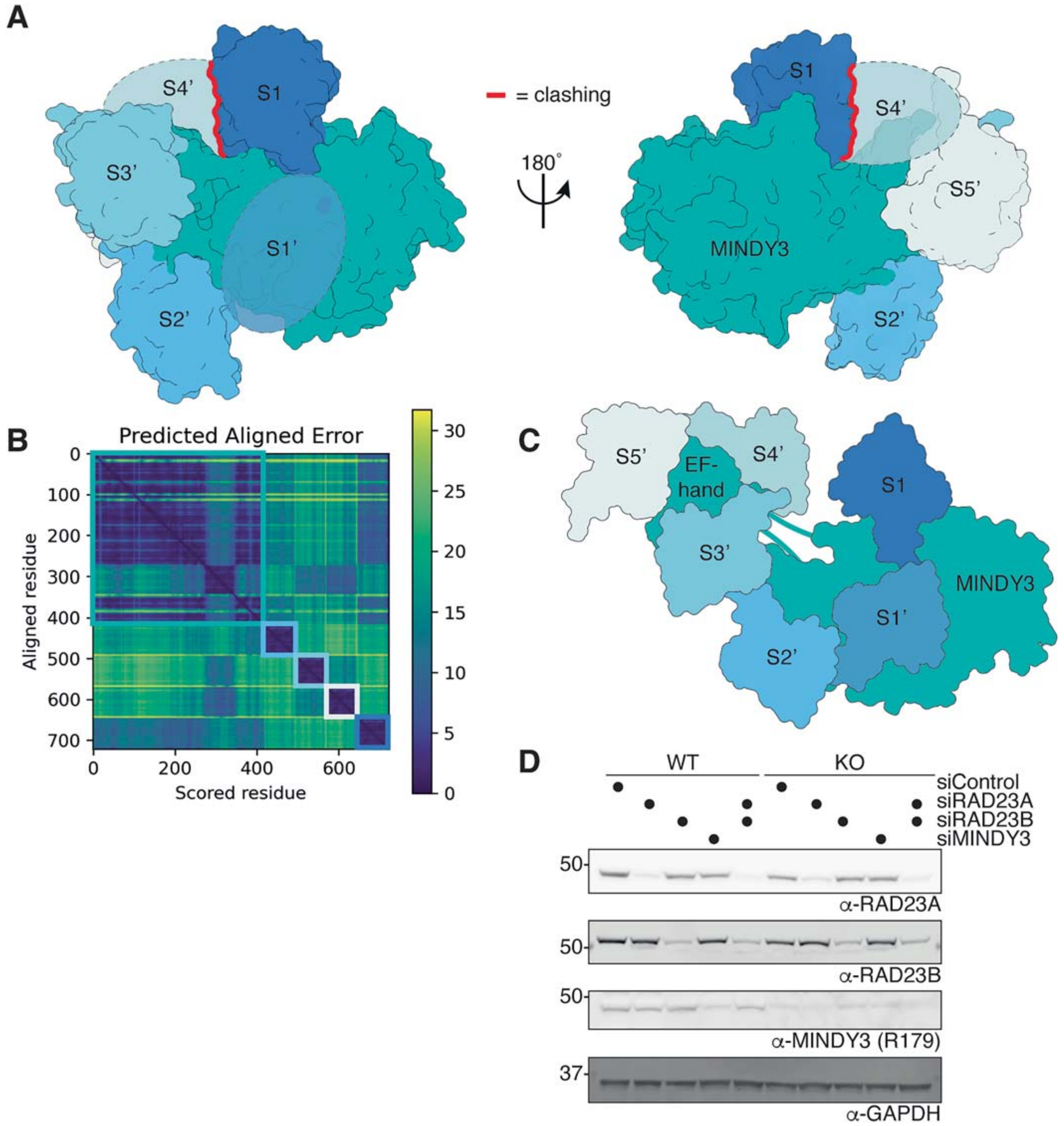

Figure EV5. MINDY3$^{EF\text{-}hand}$ is a novel ubiquitin-binding domain facilitating interaction with longer chain substrates.

(**A**) AlphaFold model of MINDY3 with four ubiquitin molecules where Ub is placed at the S1, S2′, S3′ and S5′ sites. A ubiquitin can be placed at the putative S1′ site based on geometry and the distance between the S1 and S2′ ubiquitin molecules. The S3′ and S5′ ubiquitin molecules correspond to the distal and proximal ubiquitins in the EF-hand:UBL model in Fig. 2D. Modelling the medial ubiquitin onto this AlphaFold model results in extensive clashing with the S1 ubiquitin. (**B**) PAE plot for the AlphaFold model of MINDY3 with four ubiquitin molecules. Coloured boxes indicate individual proteins as in Fig. EV5C. (**C**) Schematic of MINDY3 bound to 6 ubiquitin molecules. The core catalytic domain binds to 3 ubiquitins, whilst the EF-hand binds the remaining 3. The steric clash between the S4′ and S1 ubiquitins seen in (Fig. EV5A) is avoided by repositioning the EF-hand via the flexible linkers that connect it to the core catalytic domain. (**D**) MINDY3 and RAD23A/B do not affect each other's abundance. WT and MINDY3 KO RPE-1 cells were transfected with siRNAs targeting MINDY3, RAD23A or RAD23B, and protein levels assessed via western blot using the indicated antibodies.

© The Author(s)
