# Supplementary material for: The EF-hand domain of MINDY3 is a ubiquitin and RAD23 UBL-binding domain
Source: EMBO Rep. 2026 Jun 9;27(13):3604–31. doi: 10.1038/s44319-026-00825-1 (PMC13354579; doi:10.1038/s44319-026-00825-1)
Supplement: Supplementary file 2 — Peer Review File [file 44319_2026_825_MOESM2_ESM.pdf]

EMBO reports

The EF-hand domain of MINDY3 is a Ubiquitin and RAD23 UBL-binding domain

Yogesh Kulathu, Lee Armstrong, Matthew McFarland, Rachel O'Dea, Rościsław Krutyhołowa, Magdalena Gorka, Thomas Carroll, and Sebastian Glatt

Corresponding author(s): Yogesh Kulathu (y.kulathu@dundee.ac.uk)

Review Timeline:

Submission Date:	15th Jul 25
Editorial Decision:	2nd Sep 25
Revision Received:	31st Jan 26
Editorial Decision:	19th Mar 26
Revision Received:	11th May 26
Accepted:	27th May 26

Editor: Martina Rembold

Transaction Report:

(Note: With the exception of the correction of typographical or spelling errors that could be a source of ambiguity, letters and reports are not edited. Depending on transfer agreements, referee reports obtained elsewhere may or may not be included in this compilation. Referee reports are anonymous unless the Referee chooses to sign their reports.)

Dear Yogesh,

Thank you for the submission of your research manuscript to our journal. We have now received the two referee reports copied below.

As you will see, both referees acknowledge that the findings are interesting and that the conclusions are overall supported by the data presented but they also raise a number of concerns and have suggestions how to further strengthen the data that need to be addressed.

Given the constructive comments and support by the referees, we would like to invite you to revise your manuscript with the understanding that the referee concerns (as detailed above and in their reports) must be fully addressed and their suggestions taken on board. Please address all referee concerns in a complete point-by-point response. Acceptance of the manuscript will depend on a positive outcome of a second round of review. It is EMBO Reports policy to allow a single round of revision only and acceptance or rejection of the manuscript will therefore depend on the completeness of your responses included in the next, final version of the manuscript.

We realize that it is difficult to revise to a specific deadline. In the interest of protecting the conceptual advance provided by the work, we recommend a revision within 3 months (December 1st). Please discuss the revision progress ahead of this time with the editor if you require more time to complete the revisions.

I am also happy to discuss the revision further via e-mail or a video call, if you wish.

You can either publish the study as a short report or as a full article. For short reports, the revised manuscript should not exceed 27,000 characters (including spaces but excluding materials & methods and references) and 5 main plus 5 expanded view figures. The results and discussion sections must further be combined, which will help to shorten the manuscript text by eliminating some redundancy that is inevitable when discussing the same experiments twice. For a normal article there are no length limitations, but it should have more than 5 main figures and the results and discussion sections must be separate. In both cases, the entire materials and methods must be included in the main manuscript file.

================

IMPORTANT NOTE:

We perform an initial quality control of all revised manuscripts before re-review. Your manuscript will FAIL this control and the handling will be delayed IN CASE the following APPLIES:

1) A data availability section providing access to data deposited in public databases is missing. If you have not deposited any data, please add a sentence to the data availability section that explains that.

2) Your manuscript contains statistics and error bars based on n=2. Please use scatter blots in these cases. No statistics should be calculated if n=2.

When submitting your revised manuscript, please carefully review the instructions that follow below. Failure to include requested items will delay the evaluation of your revision.

================

When submitting your revised manuscript, we will require:

1) a .docx formatted version of the manuscript text (including legends for main figures, EV figures and tables). Please make sure that the changes are highlighted to be clearly visible.

2) individual production quality figure files as .eps, .tif, .jpg (one file per figure).
Please download our Figure Preparation Guidelines (figure preparation pdf) from our Author Guidelines pages https://www.embopress.org/page/journal/14693178/authorguide for more info on how to prepare your figures.

3) a .docx formatted letter INCLUDING the reviewers' reports and your detailed point-by-point responses to their comments. As part of the EMBO Press transparent editorial process, the point-by-point response is part of the Review Process File (RPF), which will be published alongside your paper.

4) a complete author checklist, which you can download from our author guidelines (<https://www.embopress.org/page/journal/14693178/authorguide>). Please insert information in the checklist that is also reflected in the manuscript. The completed author checklist will also be part of the RPF.

5) Please note that all corresponding authors are required to supply an ORCID ID for their name upon submission of a revised manuscript (<https://orcid.org/>). Please find instructions on how to link your ORCID ID to your account in our manuscript tracking system in our Author guidelines (<https://www.embopress.org/page/journal/14693178/authorguide#authorshipguidelines>)

6) We replaced Supplementary Information with Expanded View (EV) Figures and Tables that are collapsible/expandable online. A maximum of 5 EV Figures can be typeset. EV Figures should be cited as 'Figure EV1, Figure EV2" etc... in the text and their respective legends should be included in the main text after the legends of regular figures.

- For the figures that you do NOT wish to display as Expanded View figures, they should be bundled together with their legends in a single PDF file called *Appendix*, which should start with a short Table of Content. Appendix figures should be referred to in the main text as: "Appendix Figure S1, Appendix Figure S2" etc. See detailed instructions regarding expanded view here: <https://www.embopress.org/page/journal/14693178/authorguide#expandedview>

- Additional Tables/Datasets should be labeled and referred to as Table EV1, Dataset EV1, etc. Legends have to be provided in a separate tab in case of .xls files. Alternatively, the legend can be supplied as a separate text file (README) and zipped together with the Table/Dataset file.

7) Please note that a Data Availability section at the end of Materials and Methods is now mandatory. In case you have no data that requires deposition in a public database, please state so instead of refereeing to the database.
See also < https://www.embopress.org/page/journal/14693178/authorguide#dataavailability>). Please note that the Data Availability Section is restricted to new primary data that are part of this study.

7) Please format the "Data Availability" along these lines: "The [structural coordinates | microarray | mass spectrometry] data from this publication have been deposited to the [name of the database] database [URL] and assigned the identifier [accession | permalink | hashtag]."). It is mandatory to include a URL that resolves directly to the deposited datasets.

8) At EMBO Press we ask authors to provide source data for the main manuscript figures. You will receive a separate email with instructions for providing source data with your revised manuscript, including how to upload and organize the files.

Additional information on source data and instruction on how to label the files are available <https://www.embopress.org/page/journal/14693178/authorguide#sourcedata>

9) The journal requires a statement specifying whether or not authors have competing interests (defined as all potential or actual interests that could be perceived to influence the presentation or interpretation of an article). In case of competing interests, this must be specified in your disclosure statement. Further information: https://www.embopress.org/competing-interests

10) Figure legends and data quantification:
The following points must be specified in each figure legend:

- the name of the statistical test used to generate error bars and P values,
- the EXACT p-values,
- the number (n) of independent experiments (please specify technical or biological replicates) underlying each data point,
- the nature of the bars and error bars (s.d., s.e.m.)

- If the data are obtained from n {less than or equal to} 5, show the individual data points in addition to the SD or SEM.
- If the data are obtained from n {less than or equal to} 2, use scatter blots showing the individual data points.

Discussion of statistical methodology can be reported in the materials and methods section, but figure legends should contain a basic description of n, P and the test applied.

See also the guidelines for figure legend preparation:
https://www.embopress.org/page/journal/14693178/authorguide#figureformat

- Please also include scale bars in all microscopy images and define their size in the legend, not the image.

11) Our journal encourages inclusion of *data citations in the reference list* to directly cite datasets that were re-used and obtained from public databases. Data citations in the article text are distinct from normal bibliographical citations and should

directly link to the database records from which the data can be accessed. In the main text, data citations are formatted as follows: "Data ref: Smith et al, 2001" or "Data ref: NCBI Sequence Read Archive PRJNA342805, 2017". In the Reference list, data citations must be labeled with "[DATASET]". A data reference must provide the database name, accession number/identifiers and a resolvable link to the landing page from which the data can be accessed at the end of the reference. Further instructions are available at <https://www.embopress.org/page/journal/14693178/authorguide#referencesformat>.

12) All Materials and Methods need to be described in the main text using our 'Structured Methods' format. According to this format, the Methods section includes a Reagents and Tools Table (listing key reagents, experimental models, software and relevant equipment and including their sources and relevant identifiers) followed by a Methods and Protocols section describing the methods, ideally using a step-by-step protocol format. The aim is to facilitate adoption of the methodologies across labs. Please download and fill our Reagents and Tools Table template (.docx), which you can find in our author guidelines: https://www.embopress.org/page/journal/14693178/authorguide#structuredmethods.
When submitting your revised manuscript, please do not include the Reagents and Tools Table in the Methods section of the manuscript but upload it as a separate file choosing the file type "Reagent Table".
An example of a Method paper with Structured Methods can be found here: https://www.embopress.org/doi/10.15252/msb.20178071.

13) As part of the EMBO publication's Transparent Editorial Process, EMBO Reports publishes online a Review Process File to accompany accepted manuscripts. This File will be published in conjunction with your paper and will include the referee reports, your point-by-point response and all pertinent correspondence relating to the manuscript.

You are able to opt out of this by letting the editorial office know (emboreports@embo.org). If you do opt out, the Review Process File link will point to the following statement: "No Review Process File is available with this article, as the authors have chosen not to make the review process public in this case."

We would also welcome the submission of cover suggestions, or motifs to be used by our Graphics Illustrator in designing a cover.

I look forward to seeing a revised form of your manuscript when it is ready and please let me know if you have questions or comments regarding the revision.

Kind regards,

Martina

Martina Rembold, PhD
Senior Editor
EMBO reports

==================

Referee #1:

In the present manuscript, Armstrong et al. characterize the catalytic mechanism and binding partners of MINDY-3, an understudied deubiquitylase of the MINDY family. Using elegant biochemical and biophysical approaches, the authors demonstrate that, similar to MINDY-1/2, MINDY-3 cleaves K48-linked ubiquitin chains in a length-dependent manner, switching from exo (<5 Ub) to endo cleavage (>5 Ub). Through crystallographic, AlphaFold, and biochemical validation studies, the authors provide the structural basis for this length preference, identifying an atypical EF-hand domain inserted in the catalytic domain of MINDY-3 as a ubiquitin-binding domain harboring three ubiquitin-binding sites and required for cleaving polyubiquitin chains. Interestingly, the authors show that the atypical EF-hand also binds the UBL domain of RAD23A/B but not the UBL domains of other tested proteins. This interaction is confirmed in vitro and in cells upon crosslinking. Evidence is provided that this interaction serves to recruit MINDY-3 to sites of DNA damage. Finally, the authors show that MINDY-3, but not MINDY-2, can cleave K48-linked ubiquitin pre-bound to RAD23A, supporting a potential role in processing RAD23A-bound clients.

This is a well-done study which identifies preference for cleaving K48 linkages in a length-dependent manner as a shared feature of the MINDY DUB family. In addition, this study convincingly shows that the EF-hand domain within the catalytic domain of MINDY-3 is a previously unrecognized ubiquitin-binding module that also binds RAD23A/B proteins.

Overall, the manuscript is well written, and the experiments are of high quality and support the authors' claims. Aspects of this study that could be strengthened include the generalizability of atypical EF-hands as ubiquitin binding domains, the cellular

assays to further confirm a role for MINDY-3 in regulating DNA damage responses, as well as additional experiments exploring a role for MINDY-3 in editing RAD23A/B clients (see major points below). In addition, I raise some minor points, mostly regarding inconsistencies and clarifications that might help the reader more readily understand the experiments and figures.

Major points:

• As the authors point out, it was previously not appreciated that EF-hand domains are able to bind ubiquitin. Based on their studies here (and the residues they identify required for ubiquitin binding), could bioinformatic and biochemical approaches be used to predict and discover EF-hand domains in other proteins that bind ubiquitin? Could this represent a more general ubiquitin-binding module?
• The authors show MINDY-3 but not MINDY-3 lacking the EF-hand domain to localize to nuclear foci upon micro-irradiation (Fig. 6A). While this is indicative of MINDY-3 localizing to DNA damage sites, I think it would be important to include an appropriate DNA damage marker that co-localizes with these foci to unequivocally demonstrate this point. In addition, one could consider including MINDY-1/2 as negative controls in these experiments.
• Loss of MINDY-3 only has a minor effect in the rate of clearance of CPD lesions in U2OS cells (Fig 6D). Have the authors considered performing these experiments in the presence of ectopic expression of WT and catalytically inactive MINDY-3? Would this have stronger effects?
• The author's model that MINDY-3 can edit ubiquitin chains of RAD23A clients is largely based on in vitro assays, which convincingly show that MINDY-3, but not MINDY-2 is able to efficiently cleave K48-linked ubiquitin chains prebound to RAD23A (Figure 7B). Could the authors provide additional cellular evidence for this model? While I appreciate that identifying specific RAD23A clients may be technically difficult and beyond the scope of this manuscript, could the authors perform RAD23A/B IPs from U2OS cells upon irradiation and investigate ubiquitylation patterns? Would these patterns change in the absence of MINDY-3 or upon ectopic expression of WT or catalytic mutant MINDY-3 (and not MINDY1/2)?

Minor points:
• The authors could consider adding a section discussing how K48 linkage selectivity is achieved by MINDY-3 despite the EF-hand domain binding to K11 and K48-K63 branches
• It took me a bit of time to understand the color coding for the analytical SEC assays used throughout the study. Consider adding labels for the proteins on the side of the Coomassie gels
• Is MINDY-3 tissue or cell-type specifically expressed and if so would this help in speculating about its role in RAD23-mediated processes?
• Consider adding experimental schemes for Figure 7A/B to more readily understand the difference in prebinding RAD23 to MINDY versus RAD23 to ubiquitin
• Line 241: reference should be to Table 2
• Line 259: the proximal site is site A not C

Referee #2:

The MINDY family is a relatively recently discovered class of cysteine-protease deubiquitinases (DUBs). This DUB class selectively cleaves Lys48-linked polyubiquitin chains. Among its four members, MINDY1 and MINDY2 have been well characterized enzymatically and structurally, whereas MINDY3 has not. In this report, Armstrong et al. characterized MINDY3 and found that MINDY3 cleaves a ubiquitin (Ub) chain longer than 6 Ubs in an endo fashion and one shorter than 5 Ubs in an exo fashion from the distal end, like MINDY1 and MINDY2. They also showed that the EF-hand domain that is uniquely inserted into the catalytic domain of MINDY3 binds to a tri-Ub chain (Ub3) with micromolar affinity. This binding enables MINDY3 to cleave longer Ub chains more efficiently. Further yeast two-hybrid and in vitro binding analyses demonstrated that the EF-hand domain of MINDY3 also binds to the UBL domain of RAD23A/B, which is a shuttle factor for the proteasome and functions in DNA damage repair. The crystal structure of the MINDY3 EF-hand domain in complex with the RAD23A UBL domain revealed a structural basis for their specific binding. Overall, every experiment was appropriately performed, and the result is explicitly presented, although physiological significance of the newly identified interaction between MINDY3 and RAD23A/B remains unclear. The following points should be addressed prior to publication.

Major point:
1. The deletion analysis shows that the EF-hand domain binds to Ub3 but does not indicate that all predicted interfaces shown in Fig. 3D (i.e., Sites A, B, and C) contribute to the binding and cleavage efficiency. Even in the case using a single interface for binding to Ub, longer Ub chains can show higher affinity. To make this point clear and support the model shown in Fig. EV7, the cleavage activities of individual MINDY3 mutants at Site A, B, or C should be tested. Similar experiments for MINDY2 have been shown in Figure 5K in "Abdul Rehman, Syed Arif et al. Molecular Cell, Volume 81, Issue 20, 4176 - 4190.e6".

2. The provided PDB validation report for the apo-MINDY3 structure is incomplete, raising concerns regarding crystallography, although the statistics of the crystallography shown in Table 1 look basically fine.

3. The experiment shown in Figure 6 indicates that the recruitment of MINDY3 to DNA damage sites depends on the EF-hand domain but is not sufficient to prove RAD23A/B-dependent recruitment. The possibility of the ubiquitin chain- or other factors-

mediated recruitment cannot be excluded. To substantiate the claim that "MINDY3 is recruited to sites of DNA damage in a RAD23-dependent manner", the authors should investigate recruitment in a RAD23A/B knockout or knockdown background. Or, at least, the recruitment of a MINDY3 mutant that can bind to RAD23 but not to ubiquitin chains should be investigated.

Minor points:
1. On page 13, lines 343-350, the experiment seems to be performed in a MINDY3-knockout background, but this is not sated in the text.
2. In Table 4, the number of significant figures is inconsistent. Additionally, the resolution range reported for the refinement of MINDY3 EF-Hand+RAD23A UBL appears to be incorrect.
3. In the Materials and Methods section, the compositions of many buffers (e.g., "GST high salt buffer", "GST low salt buffer", "binding buffer", "wash buffer", "elution buffer", "GFC buffer", etc) are not described.
4. On page 3, there appear to be typographical errors in the citation for Figure 3.
5. The numbering of some tables is inconsistent with their mentions in the text.
6. The residue ranges in the PAE plots and their corresponding protein regions are unclear because the frames of the boxes indicating the regions are too thin. These should be made more prominent for clarity.

We thank the reviewers for their interest in our work, and their insightful comments and suggestions. A point-by-point response addressing these is detailed below:

Referee #1:

In the present manuscript, Armstrong et al. characterize the catalytic mechanism and binding partners of MINDY-3, an understudied deubiquitylase of the MINDY family. Using elegant biochemical and biophysical approaches, the authors demonstrate that, similar to MINDY-1/2, MINDY-3 cleaves K48-linked ubiquitin chains in a length-dependent manner, switching from exo (<5 Ub) to endo cleavage (>5 Ub). Through crystallographic, AlphaFold, and biochemical validation studies, the authors provide the structural basis for this length preference, identifying an atypical EF-hand domain inserted in the catalytic domain of MINDY-3 as a ubiquitin-binding domain harboring three ubiquitin-binding sites and required for cleaving polyubiquitin chains. Interestingly, the authors show that the atypical EF-hand also binds the UBL domain of RAD23A/B but not the UBL domains of other tested proteins. This interaction is confirmed in vitro and in cells upon crosslinking. Evidence is provided that this interaction serves to recruit MINDY-3 to sites of DNA damage. Finally, the authors show that MINDY-3, but not MINDY-2, can cleave K48-linked ubiquitin pre-bound to RAD23A, supporting a potential role in processing RAD23A-bound clients.

This is a well-done study which identifies preference for cleaving K48 linkages in a length-dependent manner as a shared feature of the MINDY DUB family. In addition, this study convincingly shows that the EF-hand domain within the catalytic domain of MINDY-3 is a previously unrecognized ubiquitin-binding module that also binds RAD23A/B proteins.

Overall, the manuscript is well written, and the experiments are of high quality and support the authors' claims. Aspects of this study that could be strengthened include the generalizability of atypical EF-hands as ubiquitin binding domains, the cellular assays to further confirm a role for MINDY-3 in regulating DNA damage responses, as well as additional experiments exploring a role for MINDY-3 in editing RAD23A/B clients (see major points below). In addition, I raise some minor points, mostly regarding inconsistencies and clarifications that might help the reader more readily understand the experiments and figures.

Major points:

• As the authors point out, it was previously not appreciated that EF-hand domains are able to bind ubiquitin. Based on their studies here (and the residues they identify required for ubiquitin binding), could bioinformatic and biochemical approaches be used to predict and discover EF-hand domains in other proteins that bind ubiquitin? Could this represent a more general ubiquitin-binding module?

We thank the reviewer for the suggestion. We performed a DALI search against the human AlphaFold database using the MINDY3 EF hand and also sequence-based searches. The highest-scoring hits corresponded exclusively to proteins with canonical EF hands, none of which contained the key ubiquitin binding residues – or residues with equivalent propoerties - that we identified. As USP32 is the only other DUB reported to contain an EF-hand, we examined it in more detail. However, it too lacks these critical binding residues, consistent with it having a canonical rather than a Ub-binding EF-hand.

To address this point in the manuscript, we have included an excerpt of the DALI output showing the MINDY3 EF hand structurally aligned against the top 20 hits and USP32 in **Fig. EV7E**.

• The authors show MINDY-3 but not MINDY-3 lacking the EF-hand domain to localize to nuclear foci upon micro-irradiation (Fig. 6A). While this is indicative of MINDY-3 localizing to DNA damage sites, I think it would be important to include an appropriate DNA damage marker that co-localizes with these foci to unequivocally demonstrate this point. In addition, one could consider including MINDY-1/2 as negative controls in these experiments.

To address this point, we have introduced an mCherry-tagged XRCC1 construct into the existing cell lines and repeated the experiment, observing similar results to our original laser stripe. With these cells, there is some weak recruitment of the ∆EF hand mutant to damage sites, but this remains substantially below that of the wild-type protein. As suggested by the reviewer, we also took the opportunity to test the remaining human MINDY family members (MINDYs -1,-2 and -4) and saw little to no recruitment to damage sites, suggesting the recruitment to sites of DNA damange is unique to MINDY3. We have included this additional data in **Fig. EV6E.**

• Loss of MINDY-3 only has a minor effect in the rate of clearance of CPD lesions in U2OS cells (Fig 6D). Have the authors considered performing these experiments in the presence of ectopic expression of WT and catalytically inactive MINDY-3? Would this have stronger effects?
We thank the reviewer for this suggestion. We have attempted to repeat these experiments in MINDY3 KO cells rescued with wild-type or C51A MINDY3. However, while the cells over-expressing wild-type MINDY3 exhibited lower CPD intensities across the time course, these differences were not statistically significant, and the high variability observed in the C51A-expressing cells caution us against drawing a firm conclusion. A representative dot blot and quantified data from 4 independent experiments is presented below.

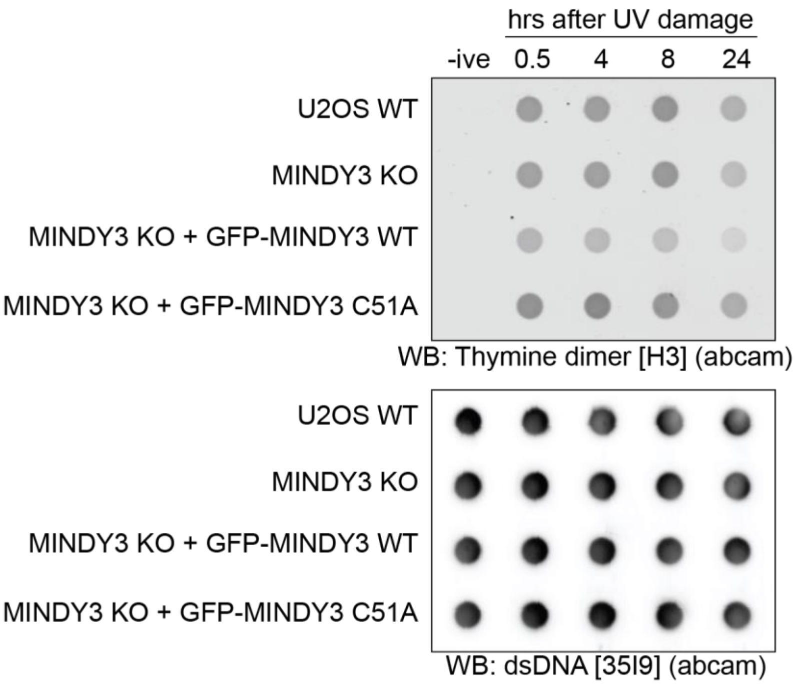

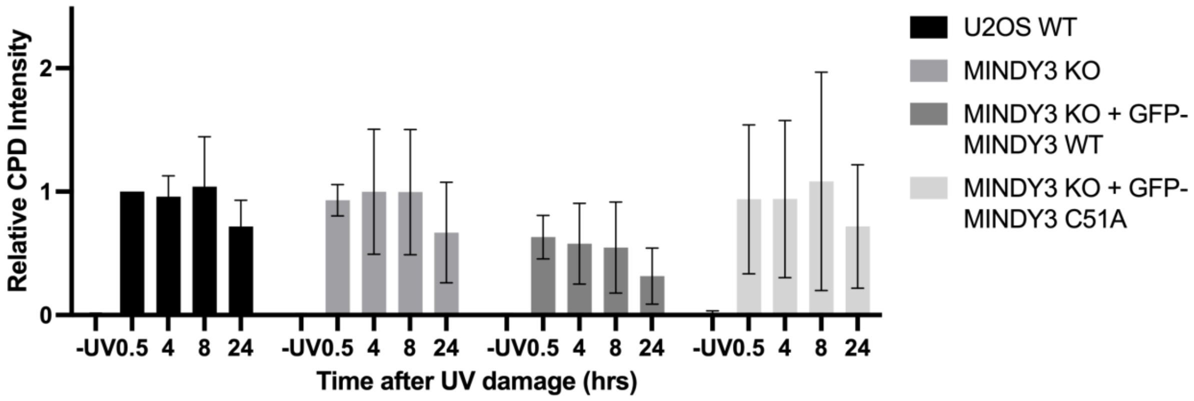

• The author's model that MINDY-3 can edit ubiquitin chains of RAD23A clients is largely based on in vitro assays, which convincingly show that MINDY-3, but not MINDY-2 is able to efficiently cleave K48-linked ubiquitin chains prebound to RAD23A (Figure 7B). Could the authors provide additional cellular evidence for this model? While I appreciate that identifying specific RAD23A clients may be technically difficult and beyond the scope of this manuscript, could the authors perform RAD23A/B IPs from U2OS cells upon irradiation and investigate ubiquitylation patterns? Would these patterns change in the absence of MINDY-3 or upon ectopic expression of WT or catalytic mutant MINDY-3 (and not MINDY1/2)?

We thank the reviewer for this suggestion. We have attempted to investigate how manipulating MINDY3 levels in cells affects the ubiquitylation of RAD23A/B-bound material after UV damage. Cells were transfected with FLAG-tagged RAD23A or RAD23B, exposed to UV damage or treated with proteasome inhibitors. RAD23 interacting proteins were isolated by co-immunoprecipitation with anti-FLAG beads,

and ubiquitylation patterns compared by western blotting. Unfortunately, we were unable to obtain conclusive results from these experiments. The wild-type control cells had a higher transfection efficiency than our MINDY3 KO and stable expression cell lines, resulting in higher expression of the FLAG-RAD23 constructs and skewing the amount of ubiquitin recovered (as seen in the example blots below).

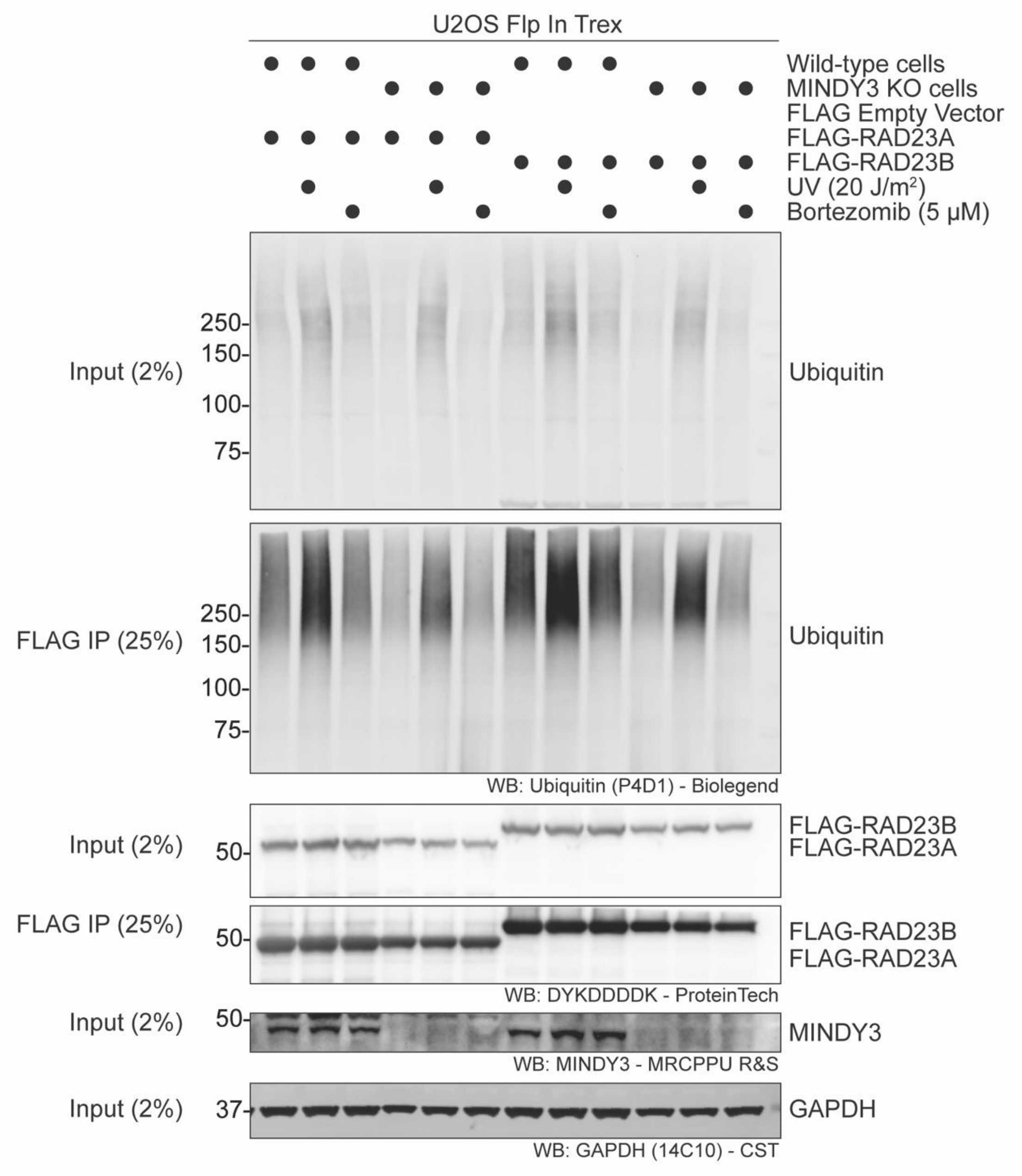

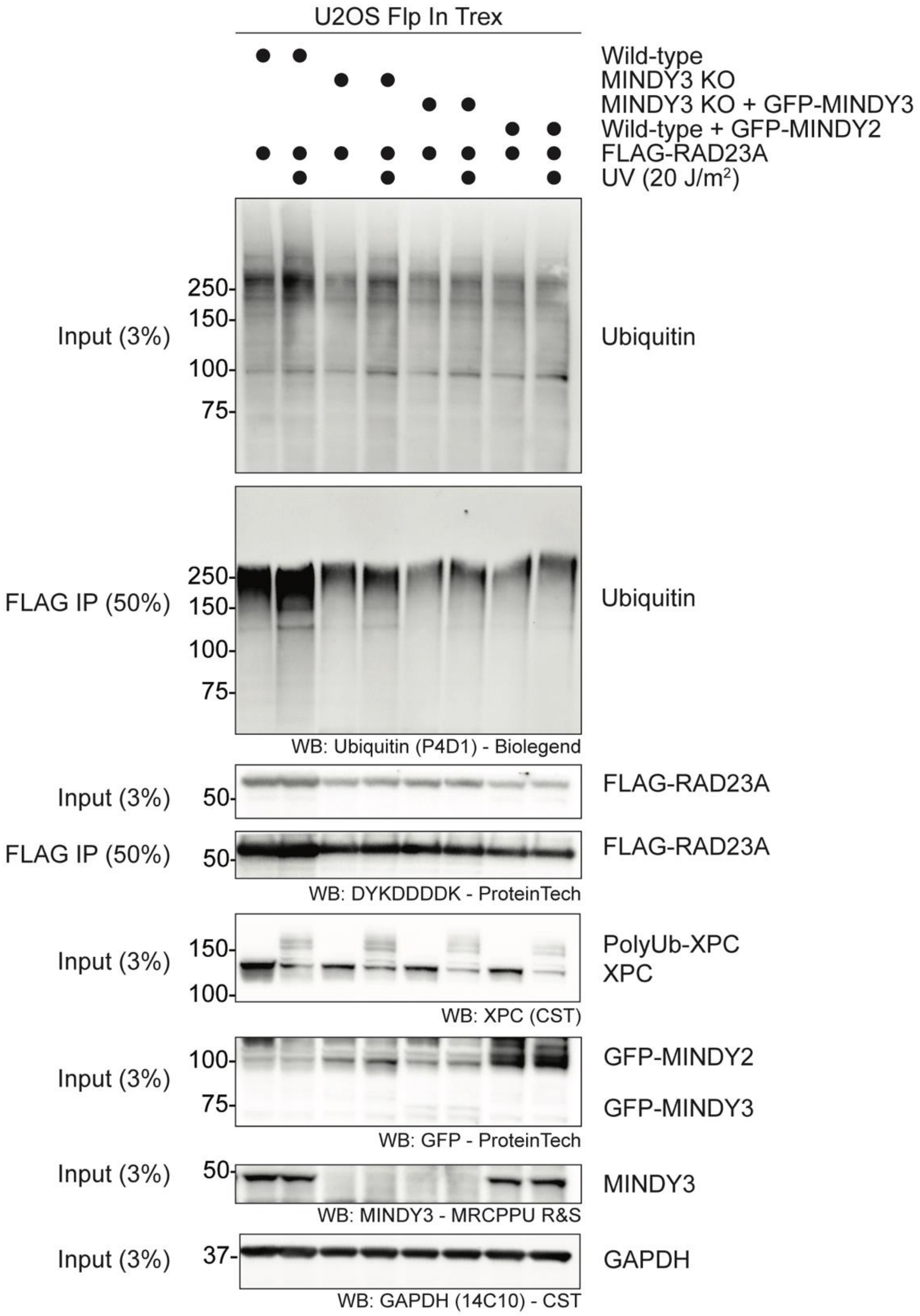

We attempted to normalise the ubiquitin signals to the amount of FLAG-RAD23A/B in the IP fractions, but the results were variable and we were unable to draw a firm conclusion as to the effect of MINDY3 on RAD23A/B-bound clients in cells with this experimental set-up.

Minor points:
• The authors could consider adding a section discussing how K48 linkage selectivity is achieved by MINDY-3 despite the EF-hand domain binding to K11 and K48-K63 branches
• It took me a bit of time to understand the color coding for the analytical SEC assays used throughout the study. Consider adding labels for the proteins on the side of the Coomassie gels
We have now added labels to make it easier to understand.
• Is MINDY-3 tissue or cell-type specifically expressed and if so would this help in speculating about its role in RAD23-mediated processes?

Analysing data available on the Human Protein Atlas, MINDY3 shows low specificity across cell and tissue types and is broadly expressed.

• Line 241: reference should be to Table 2

This has been amended.

• Line 259: the proximal site is site A not C
This has been amended.

Referee #2:

The MINDY family is a relatively recently discovered class of cysteine-protease deubiquitinases (DUBs). This DUB class selectively cleaves Lys48-linked polyubiquitin chains. Among its four members, MINDY1 and MINDY2 have been well characterized enzymatically and structurally, whereas MINDY3 has not. In this report, Armstrong et al. characterized MINDY3 and found that MINDY3 cleaves a ubiquitin (Ub) chain longer than 6 Ubs in an endo fashion and one shorter than 5 Ubs in an exo fashion from the distal end, like MINDY1 and MINDY2. They also showed that the EF-hand domain that is uniquely inserted into the catalytic domain of MINDY3 binds to a tri-Ub chain (Ub3) with micromolar affinity. This binding enables MINDY3 to cleave longer Ub chains more efficiently. Further yeast two-hybrid and in vitro binding analyses demonstrated that the EF-hand domain of MINDY3 also binds to the UBL domain of RAD23A/B, which is a shuttle factor for the proteasome and functions in DNA damage repair. The crystal structure of the MINDY3 EF-hand domain in complex with the RAD23A UBL domain revealed a structural basis for their specific binding. Overall, every experiment was appropriately performed, and the result is explicitly presented, although physiological significance of the newly identified interaction between MINDY3 and RAD23A/B remains unclear. The following points should be addressed prior to publication.

Major point:
1. The deletion analysis shows that the EF-hand domain binds to Ub3 but does not indicate that all predicted interfaces shown in Fig. 3D (i.e., Sites A, B, and C) contribute to the binding and cleavage efficiency. Even in the case using a single interface for binding to Ub, longer Ub chains can show higher affinity. To make this point clear and support the model shown in Fig. EV7, the cleavage activities of individual MINDY3 mutants at Site A, B, or C should be tested. Similar experiments for MINDY2 have been shown in Figure 5K in "Abdul Rehman, Syed Arif et al. Molecular Cell, Volume 81, Issue 20, 4176 - 4190.e6".

We have generated single point mutations in sites A (L346R), B (Y315R) and C (I358R) of the EF hand and repeated our long chain DUB assay. Intriguingly, we find that disruption of site C inhibits long chain cleavage activity, while mutations to sites A and B show similar activity to the wild-type protein. As site C is the most distal ubiquitin binding site on the EF hand, i.e. that closest to those on catalytic domain, it may be more important for positioning longer chains for efficient cleavage. We have added this data to **Figure EV4**.

2. The provided PDB validation report for the apo-MINDY3 structure is incomplete, raising concerns regarding crystallography, although the statistics of the crystallography shown in Table 1 look basically fine

We apologise that in error only the pre-validation report was provided with the initial submission - the full wwPDB validation report “for manuscript review” is now attached. Please note, that we also took the opportunity to re-refine and slightly optimize the atomic model, which is now provided with the submission. This re-evaluation has led to small changes in the statistics presented in the crystallographic table. The deposition of the model and the dataset have been completed and curated by the PDB staff – the entry (PDB ID 9RMD) is ready for release upon publication.

3. The experiment shown in Figure 6 indicates that the recruitment of MINDY3 to DNA damage sites depends on the EF-hand domain but is not sufficient to prove RAD23A/B-dependent recruitment. The possibility of the ubiquitin chain- or other factors-mediated recruitment cannot be excluded. To substantiate the claim that "MINDY3 is recruited to sites of DNA damage in a RAD23-dependent manner", the authors should investigate recruitment in a RAD23A/B knockout or knockdown background. Or, at least, the recruitment of a MINDY3 mutant that can bind to RAD23 but not to ubiquitin chains should be investigated.

We agree that this is an important experiment. However, this requires complete depletion of both RAD23 proteins. We have repeated our laser stripe recruitment assay for GFP-MINDY3 in the context of RAD23A/B double knockdown but

unfortunately were unable to see a reduction in recruitment (seen in the below graph from the relative changes in average stripe contrast after laser stripe). Based on our immunoblotting data (figure EV7), we do not get complete depletion of the two RAD23 proteins. This relative knockdown efficiency will vary across the population, so we suspect there will be a range of RAD23A/B levels in the cells targeted for laser irradiation, complicating the resulting analysis.

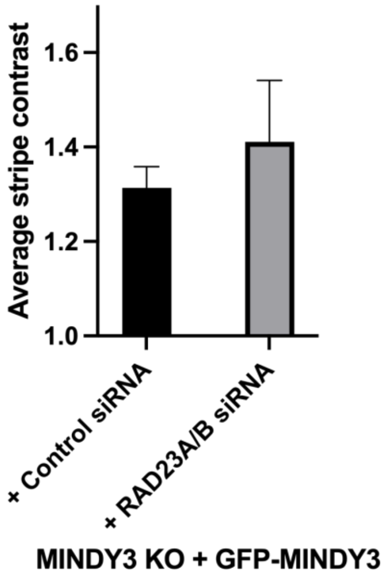

As we were unable to conclusively demonstrate a RAD23A/B dependency on MINDY3 recruitment to damage sites, we have removed reference to this claim in the text and from the title for figure 6.

Minor points:
1. On page 13, lines 343-350, the experiment seems to be performed in a MINDY3-knockout background, but this is not sated in the text.

This has now been clarified in the text.

2. In Table 4, the number of significant figures is inconsistent. Additionally, the resolution range reported for the refinement of MINDY3 EF-Hand+RAD23A UBL appears to be incorrect.

This has now been corrected.

3. In the Materials and Methods section, the compositions of many buffers (e.g., "GST high salt buffer", "GST low salt buffer", "binding buffer", "wash buffer", "elution buffer", "GFC buffer", etc) are not described.

This has now been remedied in the methods section.

4. On page 3, there appear to be typographical errors in the citation for Figure 3.

Citations have now been corrected.

5. The numbering of some tables is inconsistent with their mentions in the text.

This has now been corrected.

6. The residue ranges in the PAE plots and their corresponding protein regions are unclear because the frames of the boxes indicating the regions are too thin. These should be made more prominent for clarity.

We have made this more prominent now

Dear Yogesh,

Thank you for the submission of your revised manuscript to EMBO reports. We have now received the full set of referee reports that is copied below.

As you will see, both referees are very positive about the study and request only minor changes to clarify text and figures.

From the editorial side, there are also a few things that we need before we can proceed with the official acceptance of your study.

- I would like to publish your study in our Reports section but this would require that you do not have more than 5 main figures. Could this be arranged? The Results and Discussion sections are combined, which is perfect for Reports, but the header Discussion and future perspectives would need to be removed. For full articles, the Results and Discussion sections are separate.

- First author Lee A. Armstrong seems to be affiliated with a biotech company. Plese note that the employment in a biotech company should be stated in Declaration of Competing Interests statement.

- Regarding the Author Contributions, we now use CRediT to specify the contributions of each author in the journal submission system. CRediT replaces the author contribution section, which therefore needs to be removed from the manuscript text. You can use the free text box in our system if you wish to provide more detailed descriptions. See also guide to authors https://www.embopress.org/page/journal/14693178/authorguide#authorshipguidelines.

- Figure 6C-E: please show the individual datapoints in addition to the error bars.

- Please note that our editorial policy does not allow to base conclusions on "data not shown". Please either remove the conclusion on monoUb binding on page 8 or support it with the relevant data.

- Please add a callout for Fig. 5D in the text where appropriate.

- Please provide descriptive titles for the EV figures in the figure legends.

- Source data (microscopy images) for Figure 6B are missing. Could you please provide these as well? Thank you.

- Materials and Methods should be Methods.

- Douring our routine image checks we noticed the following issues that need your attention. Please carefully review these and clarify:

A) Figure 4C and Figure 7C
The MINDY3 and RAD23A gels shown in Figure 4C and 7C look very similar.
The source data image provided for MINDY3 for these two panels looks also similar but flipped along the Y axis.
The source data provided for RAD23A appears to be the same for 4C and 7C.
The source data "complex_raw_gel" for Figure 4C matches but is flipped relative to the panel. Please make sure that the display and orientation in the panel is the correct one.
The "Complex_raw_gel" source data file for Figure 7C does not match the respective bottom panel shown in Figure 7C.

Please check the respective files and figure composition. In case the reuse is justified because the data are from the same experiment but shown in different figure panels, please clearly specifiy this in the respective figure legends.

B) Possible reuse of gel images within Figure 5C
- The MINDY3 gels and the RAD23A gels appear to be the same in all three subpanels but the bottom panels (complex raw gels) are different in all three. This is also reflected in the raw source data files. Please check these panels and raw files. Again, please clarify whether the potential reuse is justified.

C) The four MINDY3 gels shown in Figure EV1A also appear to be the same.

- Please provide specific URLs for the deposited datasets in the Data Availability section (both for the structures and for the dataset on Zenodo).

- Please address the following points in the figure legends:
1) Define the annotated p values ****/***/**/* as well as provide the exact p-values for the same in the legend of figure 6D as

appropriate.
2) Indicate the statistical test used for data analysis in the legend of figure 6D
3) Define the error bars in the legends of figures 6C-E; EV6 B
4) Define the measure of center for the error bars in the legend of figure EV6 C

- Please add the following fund in the online manuscript tracking system: ERC Consolidator grant (grant 101002428).

- Finally, EMBO Reports papers are accompanied online by
A) a short (1-2 sentences) summary of the findings and their significance,
B) 2-3 bullet points highlighting key results and
C) a schematic summary figure that provides a sketch of the major findings (not a data image).
Please provide the summary figure as a separate file in PNG or JPG format at a size of 550x300-600 pixels (width x height). Please note that the size is rather small and that text needs to be readable at the final size. Please send us this information along with the revised manuscript.

We look forward to seeing a final version of your manuscript as soon as possible.

With kind regards,

Martina

Martina Rembold, PhD
Senior Editor
EMBO reports

==================

Referee #1:

In their revised manuscript, Armstrong et al. have performed additional experiments that directly address the points raised. The study is carefully executed and provides important mechanistic insight into MINDY3 function, including the identification of the EF-hand domain as a ubiquitin- and RAD23-binding module.
In particular, they now provide new evidence that MINDY3 localizes to sites of DNA damage in an EF-hand- domain dependent manner and show that this property is unique among MINDY family DUBs.
The authors also made clear efforts to further explore functions of MINDY3 in CPD lesion clearance and explore potential changes in RAD23-associated ubiquitylation patterns in cells. While these experiments did not yield conclusive results, the limitations are transparently discussed, and it is evident that these question is not readily feasible with the current approaches.
Overall, these additions significantly strengthen the manuscript, and I recommend it for publication.
As a minor suggestion, it would be helpful to label XRCC1 (mCherry) in EV6E to make clear that this serves as the DNA damage control marker.

Referee #2:

All my concerns, except for the following points, have been properly addressed. I recommend publication once the authors have rechecked and edited them.

1. In the Materials and Methods section, please describe the compositions of GST_PD and SEC_2 buffers.

2. In the figure legend of Fig. 3, "(F-I)" and "(I)" may be "(F-H)" and "(H)", respectively.

3. Fig. EV4F is not cited.

4. In Table 1, the space group of MINDY3 native differs from that of MINDY3 SeMet. Is it correct? In the initial manuscript, both were $P3_222$, but should be incorrect according to the attached PDB validation report ($P3_221$). Regarding this, please italicize the first letter "P" and remove the space group number. The space group of MINDY3EF-Hand+RAD23AUBL should be "$P2_1$".

We again thank the reviewers for their time and their interest in our work. A point-by-point response to both their comments and to the editorial queries is detailed below:

Dear Yogesh,

Thank you for the submission of your revised manuscript to EMBO reports. We have now received the full set of referee reports that is copied below.

As you will see, both referees are very positive about the study and request only minor changes to clarify text and figures.

From the editorial side, there are also a few things that we need before we can proceed with the official acceptance of your study.

- I would like to publish your study in our Reports section but this would require that you do not have more than 5 main figures. Could this be arranged? The Results and Discussion sections are combined, which is perfect for Reports, but the header Discussion and future perspectives would need to be removed. For full articles, the Results and Discussion sections are separate.

The manuscript has been restructured to 5 main figures and 5 accompanying EV figures as requested. Other panels have been moved to new supplementary figures.

- First author Lee A. Armstrong seems to be affiliated with a biotech company. Plese note that the employment in a biotech company should be stated in Declaration of Competing Interests statement. Done.

- Regarding the Author Contributions, we now use CRediT to specify the contributions of each author in the journal submission system. CRediT replaces the author contribution section, which therefore needs to be removed from the manuscript text. You can use the free text box in our system if you wish to provide more detailed descriptions. See also guide to
authors https://www.embopress.org/page/journal/14693178/authorguide#authorshipguidelines.

Done.

- Figure 6C-E: please show the individual datapoints in addition to the error bars. Done.

- Please note that our editorial policy does not allow to base conclusions on "data not shown". Please either remove the conclusion on monoUb binding on page 8 or support it with the relevant data.

As this is a very minor point, we have removed the conclusion as requested.

- Please add a callout for Fig. 5D in the text where appropriate. – Done.

- Please provide descriptive titles for the EV figures in the figure legends. – Done.

- Source data (microscopy images) for Figure 6B are missing. Could you please provide these as well? Thank you. –

Due to the size of the files involved, the source data for figure 6B (now fig 4E) has been deposited to Zenodo (https://doi.org/10.5281/zenodo.18809279). This has also been linked in the Data Availability section.

- Materials and Methods should be Methods. Done.

- Douring our routine image checks we noticed the following issues that need your attention. Please carefully review these and clarify:

A) Figure 4C and Figure 7C
The MINDY3 and RAD23A gels shown in Figure 4C and 7C look very similar.

A note to indicate these control panels are shared between the two figures has been added to the legend of figure 7C (now figure 5C).
The source data image provided for MINDY3 for these two panels looks also similar but flipped along the Y axis. – Corrected
The source data provided for RAD23A appears to be the same for 4C and 7C. - A note to indicate these control panels are shared between the two figures has been added to the legend of figure 7C (now figure 5C).
The source data "complex_raw_gel" for Figure 4C matches but is flipped relative to the panel. Please make sure that the display and orientation in the panel is the correct one. Corrected (now figure 3C).
The "Complex_raw_gel" source data file for Figure 7C does not match the respective bottom panel shown in Figure 7C. Corrected (now figure 5C).

Please check the respective files and figure composition. In case the reuse is justified because the data are from the same experiment but shown in different figure panels, please clearly specifiy this in the respective figure legends.

B) Possible reuse of gel images within Figure 5C
- The MINDY3 gels and the RAD23A gels appear to be the same in all three subpanels but the bottom panels (complex raw gels) are different in all three. This is also reflected in the raw source data files. Please check these panels and raw files. Again, please clarify whether the potential reuse is justified.

This figure is now EV4G. Due to an error during figure construction, the same MINDY3 mutant gel had been mistakenly used for all three panels. This figure has now been replaced with that of a replicate experiment for which the full raw data is available. A note has been added to the legend to indicate that the RAD23A control gel is the same across all conditions.

C) The four MINDY3 gels shown in Figure EV1A also appear to be the same.

Note added to EV1A legend to state the MINDY3 control is common to all conditions.

- Please provide specific URLs for the deposited datasets in the Data Availability section (both for the structures and for the dataset on Zenodo). Done.

- Please address the following points in the figure legends:
1) Define the annotated p values ****/***/**/* as well as provide the exact p-values for the same in the legend of figure 6D as appropriate.
2) Indicate the statistical test used for data analysis in the legend of figure 6D
3) Define the error bars in the legends of figures 6C-E; EV6 B
4) Define the measure of center for the error bars in the legend of figure EV6 C

Done.

- Please add the following fund in the online manuscript tracking system: ERC Consolidator grant (grant 101002428).

- Finally, EMBO Reports papers are accompanied online by
A) a short (1-2 sentences) summary of the findings and their significance,
B) 2-3 bullet points highlighting key results and

Summary: The atypical EF-hand domain of the MINDY3 deubiquitinase is a novel ubiquitin-binding domain that enables chain-length-dependent deubiquitylation. Beyond its role in chain cleavage, the EF-hand mediates specific interaction with the UBL domains of the proteasome-shuttling and DNA repair factors RAD23A/B, recruiting MINDY3 to DNA damage sites.

- The MINDY3$^{EF\text{-}hand}$ domain binds K48-linked polyubiquitin via three distinct sites, driving preferential cleavage of long chains in a chain-length-dependent manner.
- A crystal structure of the MINDY3$^{EF\text{-}hand}$:RAD23A^{UBL} complex reveals a specific interface that mediates the interaction, which is unique to RAD23A/B and absent in other UBL-domain proteins.
- The MINDY3$^{EF\text{-}hand}$ mediates interaction with RAD23A/B in cells and recruits MINDY3 to DNA damage sites, hinting at a potential role as a regulator of DNA damage responses.

C) a schematic summary figure that provides a sketch of the major findings (not a data image).
Please provide the summary figure as a separate file in PNG or JPG format at a size of 550x300-600 pixels (width x height). Please note that the size is rather small and that text needs to be readable at the final size. Please send us this information along with the revised manuscript.

Done

We look forward to seeing a final version of your manuscript as soon as possible.

With kind regards,

Martina

Martina Rembold, PhD
Senior Editor
EMBO reports

==================

Referee #1:

In their revised manuscript, Armstrong et al. have performed additional experiments that directly address the points raised. The study is carefully executed and provides important mechanistic insight into MINDY3 function, including the identification of the EF-hand domain as a ubiquitin- and RAD23-binding module.
In particular, they now provide new evidence that MINDY3 localizes to sites of DNA damage in an EF-hand- domain dependent manner and show that this property is unique among MINDY family DUBs.
The authors also made clear efforts to further explore functions of MINDY3 in CPD lesion clearance and explore potential changes in RAD23-associated ubiquitylation patterns in cells. While these experiments did not yield conclusive results, the limitations are transparently discussed, and it is evident that these question is not readily feasible with the current approaches.
Overall, these additions significantly strengthen the manuscript, and I recommend it for publication.
As a minor suggestion, it would be helpful to label XRCC1 (mCherry) in EV6E to make clear that this serves as the DNA damage control marker.

Additional labels have now been added.

Referee #2:

All my concerns, except for the following points, have been properly addressed. I recommend publication once the authors have rechecked and edited them.

1. In the Materials and Methods section, please describe the compositions of GST_PD and SEC_2 buffers.

This information has now been included

2. In the figure legend of Fig. 3, "(F-I)" and "(I)" may be "(F-H)" and "(H)", respectively. – Done

3. Fig. EV4F is not cited. – Done

4. In Table 1, the space group of MINDY3 native differs from that of MINDY3 SeMet. Is it correct? In the initial manuscript, both were $P3_222$, but should be incorrect according to the attached PDB validation report ($P3_221$). We apologize for this and fixed the error

Regarding this, please italicize the first letter "P" and remove the space group number. The space group of MINDY3EF-Hand+RAD23AUBL should be "$P2_1$". Done

Prof. Yogesh Kulathu
University of Dundee
MRC Protein Phosphorylation and Ubiquitylation Unit
Dow Street
Dundee DD1 5EH
United Kingdom

Dear Yogesh,

I am very pleased to accept your manuscript for publication in the next available issue of EMBO reports. Thank you for your contribution to our journal.

Your manuscript will be processed for publication by EMBO Press. It will be copy edited and you will receive page proofs prior to publication. Please note that you will be contacted by Springer Nature Author Services to complete licensing and payment information.

You may qualify for financial assistance for your publication charges - either via a Springer Nature fully open access agreement or an EMBO initiative. Check your eligibility: https://link.springer.com/journal/44319/how-to-publish-with-us

Should you be planning a Press Release on your article, please notify the production team via this form in order to coordinate publication and release dates: https://forms.cloud.microsoft/e/eH4A8JVP6g

If you have any questions, please do not hesitate to contact the Editorial Office. Thank you for your contribution to EMBO Reports.

Kind regards,

Martina

Martina Rembold, PhD
Senior Editor
EMBO reports

>>> Please note that it is EMBO Reports policy for the transcript of the editorial process (containing referee reports and your response letter) to be published as an online supplement to each paper. If you do NOT want this, you will need to inform the Editorial Office via email immediately. More information is available here: https://link.springer.com/partners/embo-press/editorial-policies#Peer%20review

EMBO Press Author Checklist

Corresponding Author Name: Yogesh Kulathu
Journal Submitted to: EMBO Reports
Manuscript Number: EMBOR-2025-62336V2

USEFUL LINKS FOR COMPLETING THIS FORM
The EMBO Journal - Author Guidelines
EMBO Reports - Author Guidelines
Molecular Systems Biology - Author Guidelines
EMBO Molecular Medicine - Author Guidelines

Reporting Checklist for Life Science Articles (updated January
This checklist is adapted from Materials Design Analysis Reporting (MDAR) Checklist for Authors. MDAR establishes a minimum set of requirements in transparent reporting in the life sciences (see Statement of Task: 10.31222/osf.io/9sm4x). Please follow the journal's guidelines in preparing your
Please note that a copy of this checklist will be published alongside your article.

Abridged guidelines for figures
1. Data
The data shown in figures should satisfy the following conditions:
- the data were obtained and processed according to the field's best practice and are presented to reflect the results of the experiments in an accurate and unbiased manner.
- ideally, figure panels should include only measurements that are directly comparable to each other and obtained with the same assay.
- plots include clearly labeled error bars for independent experiments and sample sizes. Unless justified, error bars should not be shown for technical
- if n<5, the individual data points from each experiment should be plotted. Any statistical test employed should be justified.
- Source Data should be included to report the data underlying figures according to the guidelines set out in the authorship guidelines on Data

2. Captions
Each figure caption should contain the following information, for each panel where they are relevant:
- a specification of the experimental system investigated (eg cell line, species name).
- the assay(s) and method(s) used to carry out the reported observations and measurements.
- an explicit mention of the biological and chemical entity(ies) that are being measured.
- an explicit mention of the biological and chemical entity(ies) that are altered/varied/perturbed in a controlled manner.
- the exact sample size (n) for each experimental group/condition, given as a number, not a range;
- a description of the sample collection allowing the reader to understand whether the samples represent technical or biological replicates (including how many animals, litters, cultures, etc.).
- a statement of how many times the experiment shown was independently replicated in the laboratory.
- definitions of statistical methods and measures:
  - common tests, such as t-test (please specify whether paired vs. unpaired), simple χ2 tests, Wilcoxon and Mann-Whitney tests, can be unambiguously identified by name only, but more complex techniques should be described in the methods section;
  - are tests one-sided or two-sided?
  - are there adjustments for multiple comparisons?
  - exact statistical test results, e.g., P values = x but not P values < x;
  - definition of 'center values' as median or average;
  - definition of error bars as s.d. or s.e.m.

Please complete ALL of the questions below.
Select "Not Applicable" only when the requested information is not relevant for your study.

Materials

Newly Created Materials	Information included in the manuscript?	In which section is the information available? (Reagents and Tools Table, Materials and Methods, Figures, Data Availability Section)
New materials and reagents need to be available; do any restrictions apply?	Not Applicable	No restrictions

Antibodies	Information included in the manuscript?	In which section is the information available? (Reagents and Tools Table, Materials and Methods, Figures, Data Availability Section)
For **antibodies** provide the following information:<br>- Commercial antibodies: RRID (if possible) or supplier name, catalogue number and or/clone number<br>- Non-commercial: RRID or citation	Yes	Reagents and Tools table, Materials and Methods

DNA and RNA sequences	Information included in the manuscript?	In which section is the information available? (Reagents and Tools Table, Materials and Methods, Figures, Data Availability Section)
**Short novel DNA or RNA including primers, probes**: provide the sequences.	Yes	Reagents and Tools table

Cell materials	Information included in the manuscript?	In which section is the information available? (Reagents and Tools Table, Materials and Methods, Figures, Data Availability Section)
**Cell lines:** Provide species information, strain. Provide accession number in repository **OR** supplier name, catalog number, clone number, and/**OR** RRID.	Yes	Reagents and Tools table, Materials and Methods
**Primary cultures:** Provide species, strain, sex of origin, genetic modification status.	Not Applicable	
Report if the cell lines were recently **authenticated** (e.g., by STR profiling) and tested for mycoplasma contamination.	Yes	Routinely tested for mycoplasma contamination

Experimental animals	Information included in the manuscript?	In which section is the information available? (Reagents and Tools Table, Materials and Methods, Figures, Data Availability Section)
**Laboratory animals or Model organisms:** Provide species, strain, sex, age, genetic modification status. Provide accession number in repository **OR** supplier name, catalog number, clone number, **OR** RRID.	Not Applicable	
**Animal observed in or captured from the field:** Provide species, sex, and age where possible.	Not Applicable	
Please detail **housing and husbandry conditions**.	Not Applicable	

Plants and microbes	Information included in the manuscript?	In which section is the information available? (Reagents and Tools Table, Materials and Methods, Figures, Data Availability Section)
**Plants:** provide species and strain, ecotype and cultivar where relevant, unique accession number if available, and source (including location for collected wild specimens).	Not Applicable	
**Microbes:** provide species and strain, unique accession number if available, and source.	Not Applicable	

Human research participants	Information included in the manuscript?	In which section is the information available? (Reagents and Tools Table, Materials and Methods, Figures, Data Availability Section)
If collected and within the bounds of privacy constraints report on age, sex and gender or ethnicity for all study participants.	Not Applicable	

Core facilities	Information included in the manuscript?	In which section is the information available? (Reagents and Tools Table, Materials and Methods, Figures, Data Availability Section)
If your work benefited from core facilities, was their service mentioned in the acknowledgments section?	Yes	Acknowledgements

Design

Study protocol	Information included in the manuscript?	In which section is the information available? (Reagents and Tools Table, Materials and Methods, Figures, Data Availability Section)
If study protocol has been **pre-registered, provide DOI in the manuscript**. For clinical trials, provide the trial registration number **OR** cite DOI.	Not Applicable	
Report the **clinical trial registration number** (at ClinicalTrials.gov or equivalent), where applicable.	Not Applicable	

Laboratory protocol	Information included in the manuscript?	In which section is the information available? (Reagents and Tools Table, Materials and Methods, Figures, Data Availability Section)
Provide DOI OR other citation details if **external detailed step-by-step protocols** are available.	Not Applicable	

Experimental study design and statistics	Information included in the manuscript?	In which section is the information available? (Reagents and Tools Table, Materials and Methods, Figures, Data Availability Section)
Include a statement about **sample size** estimate even if no statistical methods were used.	Not Applicable	
Were any steps taken to minimize the effects of subjective bias when allocating animals/samples to treatment (e.g. **randomization procedure**)? If yes, have they been described?	Yes	In laser irradiation studies all samples were subjected to the same intensity of laser and monitored at the same timepoints as described in methods.
Include a statement about **blinding** even if no blinding was done.	Yes	Quantification of the laser irradiation studies was confirmed by an additional researcher who quantified the images with blinding.
Describe **inclusion/exclusion criteria** if samples or animals were excluded from the analysis. Were the criteria pre-established?<br><br>If sample or data points were omitted from analysis, report if this was due to attrition or intentional exclusion and provide justification.	Not Applicable	No samples were excluded from analyses
For every figure, are **statistical tests** justified as appropriate? Do the data meet the assumptions of the tests (e.g., normal distribution)? Describe any methods used to assess it. Is there an estimate of variation within each group of data? Is the variance similar between the groups that are being statistically compared?	Yes	In small data sets the data was found to be visually normally distributed and compared groups had similar variance.

Sample definition and in-laboratory replication	Information included in the manuscript?	In which section is the information available? (Reagents and Tools Table, Materials and Methods, Figures, Data Availability Section)
In the figure legends: state number of times the experiment was **replicated** in laboratory.	Yes	
In the figure legends: define whether data describe **technical or biological replicates**.	Yes	

Ethics

Ethics	Information included in the manuscript?	In which section is the information available? (Reagents and Tools Table, Materials and Methods, Figures, Data Availability Section)
Studies involving **human participants**: State details of **authority granting ethics approval** (IRB or equivalent committee(s), provide reference number for approval.	Not Applicable	
Studies involving **human participants**: Include a statement confirming that **informed consent** was obtained from all subjects and that the experiments conformed to the principles set out in the WMA Declaration of Helsinki and the Department of Health and Human Services Belmont Report.	Not Applicable	
Studies involving **human participants:** For publication of **patient photos**, include a statement confirming that consent to publish was obtained.	Not Applicable	
Studies involving experimental **animals**: State details of **authority granting ethics approval** (IRB or equivalent committee(s), provide reference number for approval. Include a statement of compliance with ethical regulations.	Not Applicable	
Studies involving **specimen and field samples:** State if relevant **permits** obtained, provide details of authority approving study; if none were required, explain why.	Not Applicable	

Dual Use Research of Concern (DURC)	Information included in the manuscript?	In which section is the information available? (Reagents and Tools Table, Materials and Methods, Figures, Data Availability Section)
Could your study fall under dual use research restrictions? Please check biosecurity documents and list of **select agents and toxins** (CDC): https://www.selectagents.gov/sat/list.htm	Not Applicable	
If you used a select agent, is the security level of the lab appropriate and reported in the manuscript?	Not Applicable	
If a study is subject to dual use research of concern regulations, is the name of the **authority granting approval and reference number** for the regulatory approval provided in the manuscript?	Not Applicable	

Reporting

The MDAR framework recommends adoption of discipline-specific guidelines, established and endorsed through community initiatives. Journals have their own policy about requiring specific guidelines and recommendations to complement MDAR.

Adherence to community standards	Information included in the manuscript?	In which section is the information available? (Reagents and Tools Table, Materials and Methods, Figures, Data Availability Section)
State if relevant guidelines or checklists (e.g., **ICMJE, MIBBI, ARRIVE, PRISMA**) have been followed or provided.	Not Applicable	
For **tumor marker prognostic studies,** we recommend that you follow the **REMARK** reporting guidelines (see link list at top right). See author guidelines, under 'Reporting Guidelines'. Please confirm you have followed these guidelines.	Not Applicable	
For **phase II and III randomized controlled trials**, please refer to the **CONSORT** flow diagram (see link list at top right) and submit the CONSORT checklist (see link list at top right) with your submission. See author guidelines, under 'Reporting Guidelines'. Please confirm you have submitted this list.	Not Applicable	

Data Availability

Data availability	Information included in the manuscript?	In which section is the information available? (Reagents and Tools Table, Materials and Methods, Figures, Data Availability Section)
Have **primary datasets** been deposited according to the journal's guidelines (see 'Data Deposition' section) and the respective accession numbers provided in the Data Availability Section?	Yes	Reagents and Tools table, Materials and methods, Data availability section
Were **human clinical and genomic datasets** deposited in a public access-controlled repository in accordance to ethical obligations to the patients and to the applicable consent agreement?	Not Applicable	
Are **computational models** that are central and integral to a study available without restrictions in a machine-readable form? Were the relevant accession numbers or links provided?	Not Applicable	
If publicly available data were reused, provide the respective **data citations in the reference list**.	Not Applicable	
